# Low Levels of Pyrethroid Resistance in Hybrid Offspring of a Highly Resistant and a More Susceptible Mosquito Strain

**DOI:** 10.1093/jisesa/ieaa060

**Published:** 2020-07-01

**Authors:** Matthew Pinch, Stacy D Rodriguez, Soumi Mitra, Yashoda Kandel, Emily Moore, Immo A Hansen

**Affiliations:** 1 Department of Biology, New Mexico State University, Las Cruces, NM; 2 Institute of Applied Biosciences, New Mexico State University, Las Cruces, NM

**Keywords:** mosquito, pyrethroid, resistance

## Abstract

The use of insecticides has been a central approach to control disease-transmitting mosquitoes for the last century. The high prevalence of pyrethroid use as public health insecticides has resulted in the evolution of pyrethroid resistance in many populations of *Aedes aegypti* (Linnaeus) (Diptera: Culicidae), throughout its global distribution range. Insecticide resistance is often correlated with an associated fitness cost. In this project, we studied the phenotypes of hybrid mosquitoes derived from crossing a pyrethroid-resistant strain of *Ae. aegypti* (Puerto Rico [PR]) with a more susceptible one (Rockefeller [ROCK]). We first sequenced and compared the *para* gene of both original strains. We then crossed males from one strain with females of the other, creating two hybrids (Puertofeller, Rockorico). We used a Y-tube choice assay to measure the attraction of these strains towards a human host. We then compared the levels of pyrethroid resistance in the different strains. We found three known resistance mutations in the *para* gene sequence of the PR strain. In our attraction assays, PR females showed lower attraction to humans, than the ROCK females. Both hybrid strains showed strong attraction to a human host. In the insecticide resistance bottle assays, both hybrid strains showed marginal increases in resistance to permethrin compared to the more susceptible ROCK strain. These results suggest that hybrids of sensitive and permethrin-resistant mosquitoes have an incremental advantage compared to more susceptible mosquitoes when challenged with permethrin. This explains the rapid spread of permethrin resistance that was observed many times in the field.

Insecticides are the most important tool to control mosquitoes and their diseases. While new strategies of mosquito population control, such as gene editing, sterile insect technique, and *Wolbachia* infection, are currently being developed and tested, chemical insecticides still represent the most important tool for the control of mosquito populations and the spread of diseases that they transmit (Mendis et al. 2009, Yakob and Walker 2016, Zheng et al. 2019). Insecticides can be classified into major categories based on the life stage on which they act (targeting eggs, larvae, or adults), and by chemical structure (Sparks and Nauen 2015). The four major categories of insecticides used to control mosquitoes are carbamates, organochlorines, organophosphates, and pyrethroids (Moyes et al. 2017). Pyrethroids are derivatives of pyrethrum, a compound isolated from *Chrysanthemum* flowers (Elliott 1977). Of these four major chemical classes, pyrethroids have become the most popular family of insecticides due to their knockdown action and low toxicity to mammals (Palmquist et al. 2012). However, inappropriate insecticide use has provided selective pressure for the evolution of resistance in populations of insects worldwide (Chareonviriyaphap et al. 2013, Vanlerberghe et al. 2017).

The yellow fever mosquito, *Aedes aegypti* (Linnaeus) (Diptera: Culicidae), is a vector of many medically important diseases, including Dengue Fever, West Nile Virus, Yellow Fever, and Zika Virus (Souza-Neto et al. 2019). The global distribution of this vector has made it a particularly important component of many insect control policies, and therefore, a frequent target of pyrethroid use around the world (Smith et al. 2016). Insecticide control programs for *Ae. aegypti* stretch back to the 1940s as militaries tried to control the spread of mosquito-borne diseases among their troops (Chen et al. 2019). From this time into the 1960s, DDT, an organochlorine, was the most commonly used insecticide. Interestingly, DDT and pyrethroids share the same mode of action (Soderlund 1995, Du et al. 2016b, Zhorov and Dong 2017), so selective pressure for resistance has been maintained on the same voltage-gated sodium channel gene in *Ae. aegypti* populations for over 60 yr. In recent decades, this pressure has begun to drive widespread resistance to pyrethroids, and resistant populations have been detected on all continents with the exception of Australia (Endersby-Harshman et al. 2017, Moyes et al. 2017). The establishment of resistance mutations in *Ae. aegypti* populations coupled with a warming climate has allowed the spread of insecticide-resistant mosquitoes across their species’ endemic range (Marcombe et al. 2009, Deming et al. 2016, Goindin et al. 2017, Moyes et al. 2017, Amelia-Yap et al. 2018, Badolo et al. 2019, Demok et al. 2019, Marcombe et al. 2019, Morales et al. 2019) and into new habitats (Kandel et al. 2019, Liebman et al. 2019), and resistant populations of a related species, *A. albopictus* detected in countries like Italy and Vietnam (Kasai et al. 2019). As the home range of *Ae. aegypti* continues to expand, more humans will be at risk for mosquito-borne diseases.

Pyrethroid resistance in *Ae. aegypti* is known to be multigenic (Donnelly et al. 2009). Known mechanisms of resistance include reductions in cuticle penetration, increased detoxification activity mediated by enzymes like cytochrome P450s, glutathione transferases, and esterases; and target site insensitivity (Kasai et al. 2014). The target of pyrethroid insecticides is the voltage-gated sodium channel (VGSC) (*para*), which is expressed in insect neurons (Soderlund 1995). They bind into distinct sites within the transmembrane pore of VGSC and block its closure. This disrupts normal action potential propagation, causing paralysis and eventually death (Du et al. 2013, Dong et al. 2014, Silver et al. 2014). Resistance mutations in the *para* gene are referred to as knockdown resistance (*kdr*) mutations, as they prevent the ‘knockdown’ paralysis that is the classical symptom of intoxication after exposure of insects to pyrethroids (Dong 2007, Dong et al. 2014).

While pyrethroid resistance provides a selective advantage for mosquitoes in areas treated extensively with pyrethroids, it may also be associated with fitness costs. Fitness costs may involve changes in normal development, morphology, mobility, and reproduction. In *Ae. aegypti*, fitness costs associated with *kdr* mutations in crosses of the pyrethroid-sensitive Rockefeller (ROCK) laboratory strain with resistant mosquitoes resulted in reduced fitness parameters, including smaller clutch sizes in resistant females, and prolonged larval development (Brito et al. 2013). Another study comparing resistant and susceptible Brazilian field populations of *Ae. aegypti* and the ROCK laboratory strain showed decreases in adult female longevity, number of egg-laying females, clutch size, and egg viability over nine generations in the resistant lineages compared to susceptible lineages (Martins et al. 2012). Additionally, emergence of female *Culex quinquefasciatus* mosquitoes was significantly lower in *kdr* mutants compared to susceptible females (Berticat et al. 2008). However, in house flies (*Musca domestica*), a particular *kdr* haplotype was shown to persist and even expand in a laboratory population in the absence of insecticide treatment over 30 generations (Rinkevich et al. 2013). Additionally, a survey of several field populations of *Ae. aegypti* in Brazil, revealed persistence of *kdr* mutations and associated pyrethroid resistance up to 10 yr after the termination of pyrethroid use (Macoris et al. 2018).

More knowledge of the inherited fitness costs and benefits of *kdr* mutations are required in order to understand how these mutations spread through *Ae. aegypti* populations, and how these *kdr* mutations may persist in resistant populations even after the removal of pyrethroid treatment. In this study, we crossed previously reported pyrethroid-resistant Puerto Rico (PR) (Reid et al. 2014) and susceptible ROCK (Brito et al. 2013, Kandel et al. 2019) laboratory strains of *Ae. aegypti* and studied the pyrethroid resistance levels and host attraction rates of the hybrid offspring compared to the parental strains.

## Methods

### Mosquito Rearing

The two mosquito strains were obtained from BEI Resources, ROCK (MRA-734) and PR (NR-48830). According to their product information sheet, BEI Resources maintains resistance in PR mosquitoes by exposing third instar larvae to 0.1 mg/ml permethrin every third generation. Mosquito rearing followed culture protocols, as seen in (Higgs 2005). Larvae were cultured in 1.5-liter pans filled with distilled water. The larvae were provided a diet of ‘Special Kitty’ pellets ad libitum. Pellets were replenished every 3 d when water was replaced. Pupae were collected daily and transferred to BugDorm-1 Insect rearing cages (Bugdorm Store, Mega View Science, Taiwan). Mosquito cages were housed in an insectary at 80% humidity and 27°C with a 14:10 (L:D) h cycle.

### Hybrid Strain Production

Hybrid strains were created through crosses using PR and ROCK strains. These strains were prepared by crossing male ROCK and female PR mosquitoes (Puertofeller) and by crossing male PR with female ROCK mosquitoes (Rockorico). The crosses were set up at a 1:1 ratio in mass with 100 males and 100 females per cage. The mosquitoes were given 48 h to mate and after this period, females were blood-fed using an artificial feeding system (Chemglass Life Sciences, Vineland, NJ). The first generation of mosquitoes from the crosses were used for all the experiments in this study.

### Para Exon Sequencing

*Aedes aegypti para* exons were retrieved from VectorBase (VectorBase Gene ID: AAEL023266; corresponding to NCBI Gene: LOC5567355). Primers for exon polymerase chain reaction (PCR) amplification were designed from intronic sequences surrounding individual exons while overlapping primers were designed to sequence multiple closely grouped exons, or large exons. See Table 1 for primer sets. Ten whole mosquitoes from each parent (PR and ROCK) strain were pooled and homogenized with a hand-held motor and pestle. Genomic DNA was extracted using a DNeasy Blood & Tissue Kit (Qiagen). PCRs were performed in an Eppendorf EPgradient Mastercycler. All PCR primers used are included in Table 1. Note that different annealing temperatures required to amplify target regions from each strain are indicated, and nested PCR was required to amplify one exon, so both ‘outer’ and ‘inner’ nested PCR primers are reported in Table 1. Amplicon size was confirmed by agarose gel electrophoresis. PCR products were cleaned up using a QIAquick PCR Purification kit (Qiagen) and sent to MCLAB (Molecular Cloning Laboratories, South San Francisco, CA) for Sanger sequencing using primers listed in Table 1. DNA sequences were analyzed using the Chromas analysis software (Technelysium, South Brisbane, Queensland). Sequences were aligned to known exon sequences (VectorBase Gene ID: AAEL023266) to facilitate mutation analysis. Exon sequences were translated using the ExPASy Translate tool from the Swiss Institute of Bioinformatics (Artimo et al. 2012) and translated exon sequences were aligned with the Para protein sequence using the Clustal Omega multiple sequence alignment tool (Madeira et al. 2019) to identify mutations.

**Table 1.** Primers used to amplify and sequence all protein-coding exons of the *para* gene

Primer	Sequence	Annealing temp. (°C)
para1_for	TCTCCTTTCTTTCCCGCAC	55.7 (PR)
para1_rev	AACACGGTCGAATCTGGTGG	58 (Rock)
para2_for	GCAAACAGCAAAACTAACTCG	55
para2_rev	CGGAAACACAAAACACAAGG	
para3_for	TTTCAGCACACCCACACACC	58
para3_rev	CAAGGCTAGTTTACTCTGCTCTGC	
para4_for	TTGAAGCCAAGTCGAGAACCG	63
para4_rev	GTTGAGATCGAATTGCGAGACC	
para5_for	GCAGAAAGCACATTTTAACCAG	55
para5_rev	CAGTTGATTATATGGGGTAAAGC	
para6_for	CGATTTGATGGATGGTTGGTG	55 (PR)
para6_rev	GTATGTGAAAAGTATGTTGGCGG	53.2 (Rock)
para7_for	GTCATTTTAGTGTTTCTCCGC	55 (PR)
para7_rev	AAAGAACATATACATTTGAGTGG	48.2 (Rock)
para8_for	ATATGTGCTTGTCGAAAGTGGC	55
para8_rev	ATGGGCAAGGTACGAAAGGG	
para9_outer_for	TTCTCGTTATCGTTTGTTTGGC	53
para9_outer_rev	TGTCTGTATATGTGAGGAAGCC	
para9_inner_for	CGAAAACAACAAACAACCACCAC	58
para9_inner_rev	GACTTAGATAATTCCTCGCTGCTC	
para10_for	AGACGAACGACCAACAACACAC	55
para10_rev	AGCCATCGCAGTTTGGAAAG	
para11_for-PR	ACAATGGCTGCGTTACTAACC	55
para11_rev-PR	TTTTCTTTGATTCATGGACAGG	
para11_for-Rock	TCTGAGTTTCATTTGATTTCGC	55
para11_rev-Rock	ATGCTTTTGGCTTTTTGTCC	
para12_for	ACACTCCTTCTAAAAACTGGCTC	55
para12_rev	ACGGTGAAATTATGGCGATG	
para13-1_for	CGCTTCTCCTTCTTCATGGTCG	58
para13-1_rev	AGTAGTACTTGGGACTCATCGC	
para13-2_for	CTGTTCATCACCCTGTGTATCG	58
para13-2_rev	CACACAACTAACGGGAACCAC	
para14_for	CAGGGGAAGACAATCAGGACAC	52
para14_rev	AATTGGGCATCTGGTGGAAG	
para15_for	GCTGCCTAAACACTCAACACC	58
para15_rev	TGCTGTAGAATCTAGAGTCCGATC	
para16-1_for	CGCTTTGGTTCAACTCATCTCC	61
para16-1_rev	ATCTTGTTCGTTTCGTTGTCGG	
para16-2_for	ACTTTTGGTCAGCCTTTCTTGC	48
para16-2_rev	TGAGTTTCTAGCGGGAATGTGG	
para17_for	AACTAGACACCACACAGAACG	50
para17_rev	ATGATAGTGATGTGAAGTCGC	
para18-1_for	ACGAATCACGAATCTCTCCTCC	56
para18-1_rev	ATCTGCTTGGTTCTTTAGGGC	
para18-2_for	AGGACGAAGTGATAGAGGACTC	48
para18-2_rev	CTTTAGGGCTTCAAGAGATAGAG	
para19_for	AATTAAACTTGGCTATCATCAACAG	55
para19_rev	CAAGCAACGGGCACAACC	
para20_for	CCTCACTCTCGTGTGTGTTACTCG	58
para20_rev	CGGGGAAGAAGAGTAGAAAATCG	
para21-1_for	CGAACTCCTGCGTTAAAGGC	58
para21-1_rev	CTTCTGCTCGTTGAAGTTGTCG	
para21-2_for	CCAGGTGGGAAAGCAGCC	58 (PR)
para21-2_rev	GCCCTATGGGTTTCAGAAGAAG	55.7 (Rock)
para22-1_for	ATTCTCGTTGACCGATGAACCC	58
para22-1_rev	TTCGTCCTCGTTGATGATACCG	
para22-2_for	TGAGGGAAGGTGACTGATTTGG	58
para22-2_rev	TGGTTCATACCCCACTTCATCC	
para22-3_for	GGCGACATGATGTTCTGTGTGG	58
para22-3_rev	TTATATGGCAGTCGGCTTTCCC	

Primer names listed do not correspond with *para* exon numbers. Primer pairs with two annealing temperatures listed show the annealing temperature necessary for amplification of the strain listed in parentheses next to that temperature. ‘Outer’ and ‘inner’ para_9 primer pairs refer to primers used in nested PCR. The outer primers were used for a first round of PCR, and products of these reactions were used as templates for a second round of PCR using the inner primers. Inner PCR reaction products and inner primers were sent for Sanger sequencing.

### Host Attraction Assays

Y-tube olfactometer bioassays were conducted to determine the overall attraction rate of each strain to the volunteer hand. The olfactometer was built similarly to the description seen in the World Health Organization guidelines (WHO 2013, Rodriguez et al. 2015). The bioassay protocol used in this study is reflective of that described in an earlier paper (Mitra et al. 2020). Briefly, 24-h-fasted mosquitoes were placed in the holding chamber and allowed to acclimate for 1 min. The volunteer placed his or her hand at either port of the ‘Y’. The mosquitoes were exposed to the hand for 1 min before the rotating doors were opened. After a total of 2 min and 45 s, the doors were closed. The number of mosquitoes in each area of the Y-tube was recorded. The percent attraction was calculated by dividing the total number of mosquitoes in the hand port by the total number of mosquitoes in the entire Y-tube. The volunteer did not shower or apply any lotions or perfumes the day of the experiment. Four experimental replicates, each with roughly 20–35 mosquitoes, were performed. This part of the study was reviewed by the Institutional Review Board at New Mexico State University (research protocol #19565 ‘Efficacy of different insect repellents’) and approved on 4 February 2020 with an expiration date of 3 February 2021.

### Permethrin Resistance Bottle Assays

The bottle assay protocol reflected that described in (Kandel et al. 2019). The protocol can be found at https://www.protocols.io/ titled ‘NMSU Mosquito Insecticide Resistance Bottle Test Protocol’. Permethrin (Catalog # 45614, CAS # 52645-53-1) was acquired from Sigma–Aldrich. The bottles were prepared 24 h prior to the experiment. After treatment, each bottle contained 86 µg of permethrin. This concentration is higher than recommended in the original CDC Bottle Bioassay protocol (Brogdon and Chan 2010, WHO 2016), which allows for a more efficient resistance test (Kandel et al. 2019). The control bottles were treated with 2 ml of acetone. ROCK, PR, Rockorico, and Puertofeller strains were tested for resistance. Nine replicates of both permethrin and control treatments were used for each of the four strains. For each replicate, 16 mosquitoes were added to a bottle containing either the experimental dose of permethrin or the control treatment. Mosquito mortality was determined by counting the number of immobile mosquitoes in 5-min intervals. Counts were continued as long as flying mosquitoes were still observed for up to 90 min post-exposure, or until 100% of the mosquitoes were immobilized.

### Statistical Analysis

Y-tube olfaction data was tested for normal distribution using a Shapiro–Wilk test. As the data was not normally distributed, we evaluated the statistical significance of the attraction rates of all four strains with a Mann–Whitney *U* test. Bottle assay data were analyzed using a Kaplan–Meier test to identify significant differences between mortality curves of hybrid and control strains. All statistical tests were performed using GraphPad software (San Diego, CA) with a significance level of α = 0.05.

## Results

### Para Gene Sequencing Reveals Differences in Amino Acid Sequence of PR and ROCK Mosquito Strains

The VectorBase Liverpool reference gene (AAEL023266) used to sequence the pyrethroid-sensitive (ROCK) (Brito et al. 2013, Kandel et al. 2019) and pyrethroid-resistant (PR) (Reid et al. 2014) laboratory strains encodes 41 exons that are organized into 13 splice variants. The first six exons, along with the first portion of exon 7, comprise the 5′ untranslated region (UTR), and the stop codon and whole 3′ UTR are contained in exon 37. Three pairs of protein-coding exons are mutually exclusive, and another protein-coding exon is only found in one splice variant. Our sequencing covered all protein-coding exons, allowing for a full profile of all mutations in all known splice variants in both laboratory strains. The reported amino acid positions correspond to the annotation from *Musca domestica*, which are compiled in a comprehensive review by Dong et al. (2014).

**Fig. 1.**
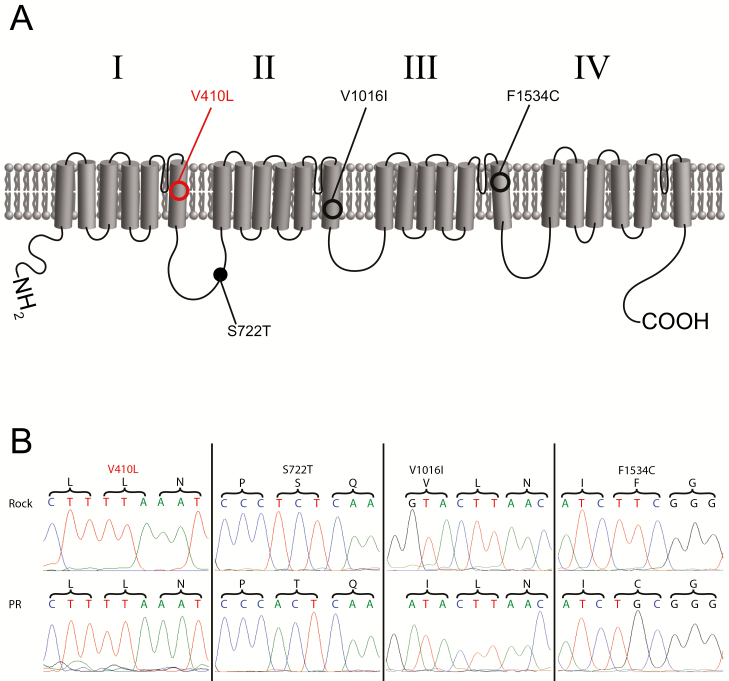
Para channel diagram with mutations from our exon sequencing. (A) The para channel consists of four transmembrane subunits (labeled I–IV), each containing six transmembrane helices. The four subunits are connected by intracellular linker regions. Mutations identified in the PR strain are marked in black, while mutations identified in both PR and ROCK strains are marked in red. Previously reported mutations associated with pyrethroid resistance in insects (Dong et al. 2014, Du et al. 2016a) are marked with open circles, while a recently reported substitution mutation (Itokawa et al. 2019, Saavedra-Rodriguez et al. 2019) found in our PR laboratory strain is marked with a filled circle. (B) Sequence chromatogram regions from ROCK and PR strains illustrating mutations and flanking nucleotide sequence (colored text). Encoded amino acids are in black text above the nucleotide sequence, with brackets marking codons. Each substitution is marked above the corresponding amino acid.

We identified three previously reported *kdr* mutations in the pyrethroid-resistant PR strain: V410L (Haddi et al. 2017), V1016I (Saavedra-Rodriguez et al. 2007), and F1534C (Kawada et al. 2009) (Fig. 1). Interestingly, we also identified the V410L substitution mutation in the ROCK strain. In addition to the previously reported *kdr* mutations in PR, we found a more recently identified non-synonymous mutation that was first reported in *Ae. aegypti* mosquitoes originating from Mexico (Itokawa et al. 2019, Saavedra-Rodriguez et al. 2019), S722T, in our PR strain (Fig. 1). This mutation is located in the intracellular linker region between subunits I and II.

Along with these reported substitution mutations, we identified other differences in the nucleotide sequence of each strain. Both strains contained several silent mutations when compared to the Liverpool reference strain (VectorBase ID: AAEL023266). PR mosquitoes contained 11 silent mutations, and ROCK mosquitoes contained 22 (Supp Data [online only]). Of these, seven silent mutations were conserved in both strains (Supp Data [online only]). We also identified a large 611 nt intronic insertion closely downstream of exon 19 in the PR strain that was not present in the ROCK sequence (Supp Data [online only]).

### Creating Hybrid Strains

The ROCK and PR strains readily crossed. Our parent strain sequence analysis allowed us to infer that the hybrid crosses would be heterozygous for substitution mutations S722T, V1016I, and F1534C, and homozygous for mutation V410L. We next performed functional studies to test the effects of these crosses on deltamethrin sensitivity and host-seeking behavior.

### Y-Tube Bioassay Reveals Significant Differences in Host Attraction Between Strains

**Fig. 2.**
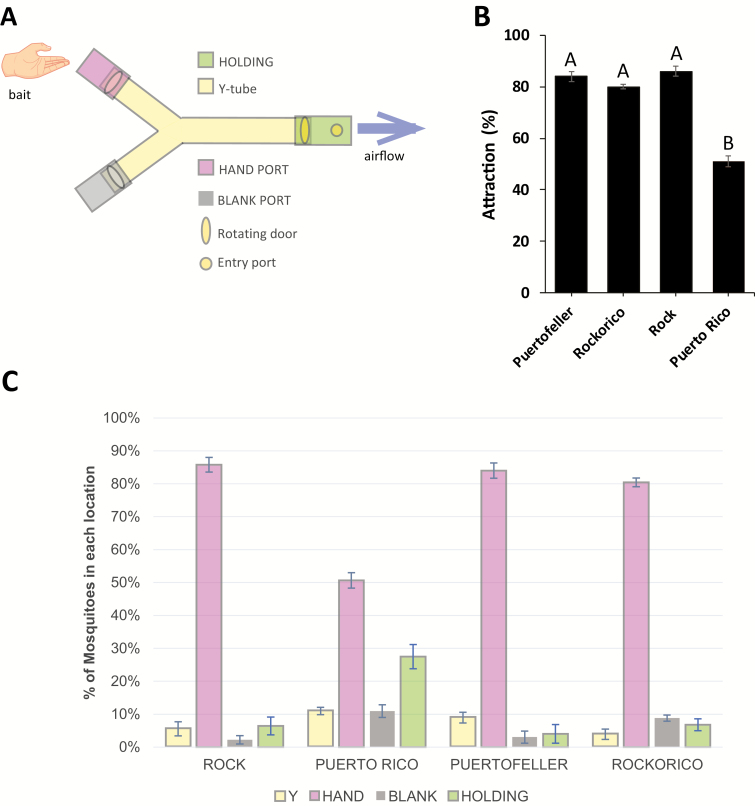
Attraction rates of pyrethroid-sensitive and -resistant parent strains and hybrid crosses. (A) schematic of Y-tube olfactometer bioassay. (B) Percent attraction of different strains analyzed in this assay. Data is presented as average percent attraction ± standard error. Letters above bars represent statistical significance categories. (C) average percent of mosquitoes from each strain found in each Y-tube chamber at the conclusion of each experiment. Data is presented as average percent of mosquitoes in each location ± standard error.

We measured host attraction in both parent strains and the two hybrid strains using a Y-tube olfactometer (Fig. 2A). ROCK mosquitoes had an average attraction rate of 86%, which was not significantly different from the Rockorico attraction rate of 80% (*P* = 0.0857), and Puertofeller attraction rate of 84% (*P* = 0.4857) (Fig. 2B). Attraction rates of the two hybrid strains were also not significantly different from each other (*P* = 0.4857). PR mosquitoes had an average attraction rate of 51%, which was significantly different from ROCK (*P* = 0.0286), Rockorico (*P* = 0.0286), and Puertofeller (*P* = 0.0286). Interestingly, the lower attraction rate observed in PR mosquitoes seems to be driven by mosquitoes that did not leave the holding chamber over the course of the bioassay (Fig. 2C).

### Permethrin Insecticide Resistance Bottle Tests Show Slight Increase in Resistance of Heterozygous Hybrid Strains

**Fig. 3.**
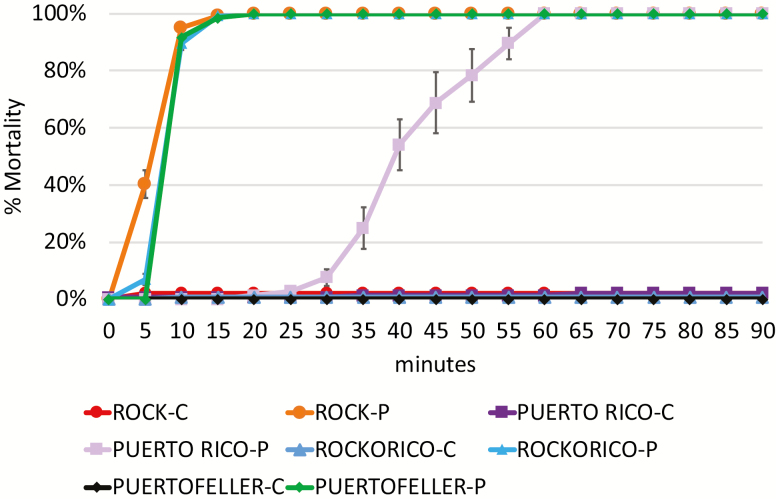
Mortality curves from permethrin bottle tests. Percent mortality for all four strains exposed to control conditions (C) or 86 µg permethrin (P) over 90 min. All data points are presented as average percent mortality ± standard error.

Mortality curves calculated from our permethrin insecticide resistance bottle assays showed that ROCK mosquitoes were extremely sensitive to permethrin having high mortality rates within the first 10 min of the assay while PR mosquitoes showed resistance with mortalities delayed for 30 min after exposure (Fig. 3). This difference in mortality rates between parent strains was statistically significant (*P* < 0.0001). Puertofeller and Rockorico percent mortality curves were also significantly different than the PR strain (*P* < 0.0001) (Fig. 3). The hybrids did show more resistance to permethrin than the ROCK strain, as there was a significant difference between mortality curves of the hybrids and the ROCK strain after 5 min of exposure (*P* < 0.0001). However, the hybrid mortality rates were no longer significantly different than the ROCK strain after 10 min of exposure. There was no significant difference between the hybrid strains across the time course of the assay (*P* = 0.2252).

## Discussion

*Aedes aegypti* mosquitoes are vectors of several medically important diseases, and as such control of their global spread and interface with human populations is of great importance. Chemical insecticides currently represent the most viable means of controlling mosquito populations. Widespread use of pyrethroid insecticides has driven the evolution of resistance in populations of *Ae. aegypti* around the world (Moyes et al. 2017). To further our understanding of how homozygous and heterozygous resistance mutations in the pyrethroid target *para* gene (Soderlund 1995) may affect fitness of female *Ae. aegypti* mosquitoes, we sequenced whole protein coding regions of the *para* gene in laboratory strains of pyrethroid-resistant PR (Reid et al. 2014), and more pyrethroid-sensitive ROCK (Brito et al. 2013, Kandel et al. 2019) mosquitoes to determine the *kdr* mutation profile in each strain. We then crossed these two strains to generate heterozygous hybrid strains (Puertofeller and Rockorico) and assayed two fitness parameters, host attraction and mortality upon pyrethroid exposure, in both PR and ROCK parent strains as well as both hybrid strains.

### The *Para* Gene of *Ae. aegypti* Laboratory Strains

Sanger sequencing revealed differences in *kdr* mutation patterns between pyrethroid-sensitive and pyrethroid-resistant strains. The PR laboratory strain used in this study contained several previously reported mutations associated with pyrethroid resistance: V410L, V1016I, and F1534C (Saavedra-Rodriguez et al. 2007, Kawada et al. 2009, Haddi et al. 2017). Each of these mutations are located in the S6 transmembrane helix of their respective subunit (V410L – I; V1016I – II; F1534C – III). Interestingly, we observed the V410L substitution in both PR and ROCK parent strains. We had selected the ROCK strain due to its previous classification as a pyrethroid susceptible strain (Brito et al. 2013, Kandel et al. 2019). However, in the presence of permethrin, ROCK mosquitoes, as well as both strain crosses had significantly truncated survival times compared to the pyrethroid-resistant PR mosquitoes (Fig. 3). This discrepancy between our sensitive mosquitoes with the V410L mutation and the previous report of resistance conferred by this mutation may be because our bottle tests used higher concentrations of permethrin (86 µg/bottle; described in detail in Kandel et al. 2019) than those in the previous study (Haddi et al. 2017). Additionally, we tested permethrin resistance in whole mosquitoes rather than by in vitro electrophysiology as performed in the previous study (Haddi et al. 2017), so the remaining *kdr* mutations missing from the ROCK *para* gene, or other physiological processes likely influenced the sensitivity of the ROCK and crossed mosquitoes to permethrin in our bottle tests.

In addition to the previously reported *kdr* mutations identified in the laboratory strains used in this study, we identified a recently reported non-synonymous mutation, S722T (Itokawa et al. 2019, Saavedra-Rodriguez et al. 2019) in our PR strain. This mutation is in the intracellular linker between subunits I and II. While it is unknown whether this mutation contributes to pyrethroid resistance, the Mexican mosquitoes in which this mutation has been detected are resistant to pyrethroid insecticides (Itokawa et al. 2019, Saavedra-Rodriguez et al. 2019). Known *kdr* mutations in this linker region are rare. Two mutations, E435K and C785R at the beginning and end of the I-II linker respectively, were identified in pyrethroid-resistant strains of the German cockroach, *Blatella germanica* (Liu et al. 2000). These mutations did not confer pyrethroid resistance in cockroaches on their own, but in combination with a known *kdr* mutation, L1014F, sensitivity to deltamethrin was increased by 500-fold (Tan et al. 2002). The S722T substitution we observed in our PR strain may also have a synergistic effect with the other observed *kdr* mutations to greatly decrease the sensitivity of Para channels to pyrethroids in these mosquitoes. Additionally, the I-II intracellular linker of the sodium VGC in mammals has phosphorylation sites that modulate sodium current (Marban et al. 1998), and cAMP/PKA-induced decreases in sodium current in *Drosophila* neurons have been proposed to be due to phosphorylation of the Para channel protein (Baines 2003). The S722T mutation may alter phosphorylation efficiency at that position in the I-II linker region and therefore affect sodium current, which may allow this mutation to contribute to resistance. Future studies will need to be conducted to determine what role this mutation may play in pyrethroid resistance in *Ae. aegypti*.

The discovery of a 611 nt insert in the intron closely 3′ to exon 19 in PR mosquitoes is also of interest. We are unsure what, if any function this insertion may have, but other studies of the para gene in *Ae. aegypti* populations have reported links between intron polymorphisms and *kdr* mutation frequencies (Martins et al. 2009, Kawada et al. 2016, Chung et al. 2019), so this insert may be associated with one of the substitution mutations we identified in the PR lab strain. Future sequencing of intronic regions may provide more insight into their associations with *kdr* mutations, which could be useful as confirmatory markers of pyrethroid resistance.

In addition to *kdr* mutations in the *para* gene, increased cytochrome P450 gene expression and activity have previously been shown to contribute to permethrin resistance in PR laboratory strain mosquitoes (Reid et al. 2014). Our study was focused on classifying the differences in *para* gene sequence between previously reported susceptible and resistant *Ae. aegypti* strains, and observing differences in fitness as measured by host attraction and pyrethroid resistance of these strains, as well as their crosses. It is likely that detoxification by P450 enzymes played a role in the observed resistance of PR mosquitoes. Future studies must be conducted to determine the expression and detoxification activities of P450 enzymes in both parent strains and hybrid crosses to develop a more complete picture of how pyrethroid resistance evolves and is maintained in populations of *Ae. aegypti*.

### Host Attraction Rates are Similar Between ROCK and Hybrid Strains

We observed a significant decrease in host attraction behavior in the pyrethroid-resistant PR lab strain when compared to the more susceptible ROCK strain and both hybrid crosses. Interestingly, this decrease in attraction was associated with a larger percentage of PR mosquitoes that did not leave the holding chamber of the olfactometer over the course of the bioassay. This result indicates that the pyrethroid resistance mutations in PR mosquitoes may have an associated fitness cost with either odor sensation or energetics necessary for flight and host-seeking behaviors. Resistance mutations in the *para* gene are known to affect the opening of neuronal sodium voltage-gated channels (Dong et al. 2014, Silver et al. 2014), which confers protection against unregulated action potential propagation caused by pyrethroid binding at the expense of altering normal action potential conductance. These alterations in neuronal signaling may affect host-seeking behavior in different ways, either by decreasing sensory neuron signaling to the central nervous system, sensory processing in the central nervous system, or by decreasing motor neuron activity thereby reducing mobility overall. It is also possible that the *kdr* mutations observed in PR mosquitoes, or other resistance mutations in other genes affects the metabolism and energetics of PR mosquitoes, thereby reducing their ability to fly. Future studies should be performed to identify causal links between known pyrethroid resistance mutations and alterations of host-seeking and flight behavior. The results of our host attraction study coupled with our mutation analysis indicate that the homozygous *kdr* phenotype in PR mosquitoes is associated with a reduction in fitness, while neither the V410L mutation alone, nor heterozygous mutations at the other positions confer reductions in host attraction.

### Heterozygous kdr Mutations Confer a Slight Increase in Pyrethroid Resistance

Our bottle test results showed an intermediate level of resistance in both heterozygous hybrid crosses when exposed to 86 µg of permethrin compared to the PR and ROCK parent strains. This intermediate resistance was observed at the 5-min time point where the mortality of both hybrid crosses was significantly lower than the ROCK strain (Fig. 3). However, by 10 min of exposure, the hybrids had similar mortality rates as the ROCK strain. These results are similar to another study conducted on hybrid crosses of pyrethroid-resistant and susceptible field populations in Thailand, which showed triple heterozygous mutants for S989P+V1016G+F1534C had increased LC_50_ for deltamethrin compared to their susceptible strain (Plernsub et al. 2016). Like that study, the crosses we observed in our study were heterozygous for three mutations (S722T+V1016I+F1534C), and homozygous for the V410L substitution. It is possible that this genotype has a fitness cost in another variables like egg-laying, metabolic energetic cost, and/or mating. When coupled with our previous results, reported above, we can conclude that under our experimental conditions, the PR/ROCK crosses containing heterozygous alleles at three points in the *para* gene have a slight fitness benefit of pyrethroid resistance without the associated fitness cost of reduced host attraction.

### Conclusions

It is well known that the spread of pyrethroid-resistant *Ae. aegypti* is an important problem for the management of *Ae. aegypti* populations worldwide (Moyes et al. 2017). However, the mechanisms of how pyrethroid resistance mutations are maintained in populations of mosquitoes even after insecticide treatments have ceased is not well understood. It has been shown that resistance mutations can persist up to 10 yr after removal of insecticide treatment in several *Ae. aegypti* populations in Brazil (Macoris et al. 2018), indicating that any fitness costs associated with pyrethroid resistance in these populations are not strong enough to select for the loss of insecticide resistance. Recently, other researchers have crossed pyrethroid-resistant and pyrethroid-sensitive field strains of *Ae. aegypti* to identify which genes are altered in heterozygous mosquitoes (Plernsub et al. 2016, Cattel et al. 2020), and the effects of heterozygous *kdr* genotypes on pyrethroid resistance (Plernsub et al. 2016). We aimed to expand on these studies by crossing pyrethroid-resistant (PR) and more susceptible (ROCK) laboratory strains and determining the fitness of the hybrid crosses in comparison to their parent strains by measuring host attraction behavior and mortality from deltamethrin exposure in all four strains. To our knowledge, this study is the first to analyze the effects of heterozygous *kdr* mutations on both pyrethroid resistance and host attraction. These data provide evidence that heterozygous individuals may serve as an important reservoir of resistance mutations for mosquito populations in the face of increased pyrethroid use, and once these mutations establish in a population, heterozygous individuals may keep these mutations circulating in that population even after the easing of insecticide use.

## Supplementary Material

ieaa060_suppl_Supplementary_Data
