# Supplementary material for: Low Levels of Pyrethroid Resistance in Hybrid Offspring of a Highly Resistant and a More Susceptible Mosquito Strain
Source: J Insect Sci. 2020 Jul 1;20(4):1. doi: 10.1093/jisesa/ieaa060 (PMC7329315; doi:10.1093/jisesa/ieaa060)
Supplement: ieaa060_suppl_Supplementary_Data [file ieaa060_suppl_supplementary_data.docx]

**Supplemental Data: Puerto Rico (resistant) & Rockefeller (more susceptible) para sequencing results.**

Exon sequences are highlighted in green, and translations of exon sequences are also provided. Notes about sequences are provided with bullet points after the translation of the corresponding sequence.

**Para1**

**>para1_PR**

TTCTCCTTTCTTTCCCGCACAGGCCTTGGAAATCTGAGGCTCTCATTGTTGGCCATATAGACAATGACCGAAGACTCCGATTCGATATCTGAGGAAGAACGTAGTTTGTTCCGTCCTTTCACCCGTGAATCATTAGCAGCAATCGAGAGACGCATCGCAGATGCCGAAGCAAAACAGCGCGAATTGGAAAAGAAGCGAGCCGAGGGGGAGGTAAGCCACCAG

atgaccgaagactccgattcgatatctgaggaagaacgtagtttgttccgtcctttcacc

M T E D S D S I S E E E R S L F R P F T

cgtgaatcattagcagcaatcgagagacgcatcgcagatgccgaagcaaaacagcgcgaa

R E S L A A I E R R I A D A E A K Q R E

ttggaaaagaagcgagccgagggggag

L E K K R A E G E

**>para1_ROCK**

TTCCCGCACAGGCCTTGGAAATCTGAGGCTCTCATTGTTGGCCATATAGACAATGACCGAAGACTCCGATTCGATATCTGAGGAAGAACGTAGTTTGTTCCGTCCTTTCACCCGTGAATCATTAGCAGCAATCGAGAGACGCATCGCAGATGCCGAAGCAAAACAGCGCGAATTGGAAAAGAAGCGAGCCGAGGGGGAGGTAAGCCACCAG

atgaccgaagactccgattcgatatctgaggaagaacgtagtttgttccgtcctttcacc

M T E D S D S I S E E E R S L F R P F T

cgtgaatcattagcagcaatcgagagacgcatcgcagatgccgaagcaaaacagcgcgaa

R E S L A A I E R R I A D A E A K Q R E

ttggaaaagaagcgagccgagggggag

L E K K R A E G E

**Para2**

**>para2_PR**

TGCAAACAGCAAAACTAACTCGAACATGTAATACATTTTTTCAGACTGGTTTTGGTCGGAAGAAAAAGAAAAAAGAAGTAAGATTTCCCACTGTTCTTCTATCATTCATTTGTTACGCTCATTTCCGTAATATCGTTATCGTTATGATTTGAGATGATTCTTTTTCGGTTACGAAACGTTTTTTTTCTGATTTTGTTTGCAATGCGATCAAACTGCGATTTTTCTAGCGAACCACAACATAAGTGCAAATCCACCTTGTGT

actggttttggtcggaagaaaaagaaaaaagaa

T G F G R K K K K K E

**>para2_ROCK**

GCAAACAGCAAAAACTAACTCGAACATGTAATACATTTTTTCAGACTGGTTTTGGTCGGAAGAAAAAGAAAAAAGAAGTAAGATTTCCCACTGTTCTTCTATCATTCATTTGTTACGCTCATTTCCGTAATATCGTTATCGTTATGATTTGAGATGATTCTTTTTCGGTTACGAAACGTTTTTTTTTTCTGGTTTTGTTTGCAATGCGATCAAACCGCGATTTTTCTAGCGAACCACAACATAAGTGCAAATCCACCTTGTGTTTTGTGTTTCCGA

actggttttggtcggaagaaaaagaaaaaagaa

T G F G R K K K K K E

**Para3**

**>para3_PR**

TTCAGCACACCCACACACCGTTGTTTTTTGAAACGTCTTCGCATTTGTGATAACCTGATTACTAATATATGATATTTTCTCTTCGTTTCGCCAATGTTTCCGTTCCATTCATACAAAACAACAGATACGCTACGACGACGAGGATGAGGATGAAGGTCCACAGCCGGATTCCACACTTGAGCAGGGAGTGCCAATCCCTGTTCGAATGCAGGGCAGCTTCCCTCCGGAATTGGCCTCCACGCCTCTCGAGGATATCGACAGCTATTACGCGAATCAGAGGGTAAGTTATTTAGCTTGGAATTAAGGAATCAGATTCAAAATGAAACTTTTGGAATATGTTGAATGTATGAAATTGGATAGAATGGAATCATGAATAAATGAAATCTAGAAGCAGCAGAGCAGAGTAAACTAGCCTTGAG

atacgctacgacgacgaggatgaggatgaaggtccacagccggattccacacttgagcag

I R Y D D E D E D E G P Q P D S T L E Q

ggagtgccaatccctgttcgaatgcagggcagcttccctccggaattggcctccacgcct

G V P I P V R M Q G S F P P E L A S T P

ctcgaggatatcgacagctattacgcgaatcagagg

L E D I D S Y Y A N Q R

**>para3_ROCK**

CCCCACACACCGTTGTTTTTTGAAACGTATTCGCATTTGTGATAACCTGATTACTAATATATGATATTTTCTCTTCGTTTCGCCAATGTTTCCGTTCCATTCATACAAAACAACAGATACGCTACGACGACGAGGATGAGGATGAAGGTCCACAGCCGGATTCCACACTTGAGCAGGGAGTGCCAATCCCTGTTCGAATGCAGGGCAGCTTCCCTCCGGAATTGGCCTCCACGCCTCTCGAGGATATCGACAGCTATTACGCGAATCAGAGGGTAAGTTATTTAACTTGGAATTAAGGAATCAGATTCAAAATGAAACTTTTGGAATATGTTGAATGTATAAAATTGGATTGAATGGAATCATGAATAAATGAAATCTAGAAGCAGCAGAGCAGAGTA

atacgctacgacgacgaggatgaggatgaaggtccacagccggattccacacttgagcag

I R Y D D E D E D E G P Q P D S T L E Q

ggagtgccaatccctgttcgaatgcagggcagcttccctccggaattggcctccacgcct

G V P I P V R M Q G S F P P E L A S T P

ctcgaggatatcgacagctattacgcgaatcagagg

L E D I D S Y Y A N Q R

**Para4**

**>para4_PR**

GAGAACCGAATTTTAATCAAAACTTTTCCCATTTCTTCTTCTTCTCTTTCCAGACATTCGTAGTAGTTAGTAAAGGAAAAGATATTTTTCGTTTCTCCGCAACCAATGCATTATATGTACTCGATCCGTTCAATCCTATACGTCGCGTAGCTATTTATATTTTAGTACATCCACTGTTTTCATTTTTTATAATAACAACCATTCTTACCAATTGTATATTGATGATCATGCCTAGCACGCCGACCGTCGAATCTACC*GA*GTAAGTATCCACTTTCTTTGTCTTATGTGAGTGTGTTTAAACAGTTTTAAGCCTTTATATTTTTCTGTAACCTGAATAATATGTCGCACTTTTCTAGAGCAGAAAGTTAGGAAACATATAACCGAACTTAGATGAGTTTTTTTTTTGGTCTCGCAATC

acattcgtagtagttagtaaaggaaaagatatttttcgtttctccgcaaccaatgcatta

T F V V V S K G K D I F R F S A T N A L

tatgtactcgatccgttcaatcctatacgtcgcgtagctatttatattttagtacatcca

Y V L D P F N P I R R V A I Y I L V H P

ctgttttcattttttataataacaaccattcttaccaattgtatattgatgatcatgcct

L F S F F I I T T I L T N C I L M I M P

agcacgccgaccgtcgaatctacc*ga*

S T P T V E S T *E*

- **Last two italicized nucleotides are part of codon spanning exon splice site (see para5 sequence below).**

**>para4_ROCK**

ACATTCGTAGTAGTTAGTAAAGGAAAAGATATTTTTCGTTTCTCCGCAACCAATGCATTATATGTACTCGATCCGTTCAATCCTATACGTCGCGTAGCTATTTATATTTTAGTACATCCACTGTTTTCATTTTTTATAATAACAACCATTCTTACCAATTGTATATTGATGATCATGCCTAGCACGCCGACCGTCGAATCTACC*GA*GTAAGTATCCACTTTCTTTGTCTTATGTGAGTGTGTTTAAACAGTTTTAAGCCTTTATATTTTTCTGTAACCTGAATAATATGTCGCACTTTTCTAGAGCAGAAAGTTAGGAAACATATAACCGAACTTAGATGAGTTTTTTTTTGGG

acattcgtagtagttagtaaaggaaaagatatttttcgtttctccgcaaccaatgcatta

T F V V V S K G K D I F R F S A T N A L

tatgtactcgatccgttcaatcctatacgtcgcgtagctatttatattttagtacatcca

Y V L D P F N P I R R V A I Y I L V H P

ctgttttcattttttataataacaaccattcttaccaattgtatattgatgatcatgcct

L F S F F I I T T I L T N C I L M I M P

agcacgccgaccgtcgaatctacc*ga*

S T P T V E S T *E*

- **Last two italicized nucleotides are part of codon spanning exon splice site (see para5 sequence below).**

**Para5**

**>para5_PR**

TTTAACCAGCTCAAAAAGCGTTGATTTTATCACCACTGTCAACTAACCGTTTTCCATTCTTCGAACCAACAACAACAACTACTACCGAAAAACCTCAG*G*GTGATATTCACCGGCATCTACACGTTCGAATCAGCTGTAAAAGTGATGGCGCGAGGTTTCATATTACAACCGTTTACTTATCTTAGAGATGCATGGAATTGGTTGGACTTCGTAGTAATAGCATTA*GC*GTGAGTTTTCCAGACACTAATCCTTATTGTGTTCGATTTTTCTTAATACTCCATATATACAATGCTTTACCCC

*g*gtgatattcaccggcatctacacgttcgaatcagctgtaaaagtgatggcgcgaggtttc

V I F T G I Y T F E S A V K V M A R G F

atattacaaccgtttacttatcttagagatgcatggaattggttggacttcgtagtaata

I L Q P F T Y L R D A W N W L D F V V I

gcatta*gc*

A L *A*

- **First italicized nucleotide is part of codon spanning exon splice site (see para4 sequence above). Last two italicized nucleotides are part of codon spanning exon splice site (see para6 sequence below).**

**>para5_ROCK**

ACATTTTAACCAGCTCAAAAGCGTTGATTTTATCACCACTGTCAACTAACCGTTTTCCATTCTTCGAACCAACAACAACTACTACTACCGAAAAACCTCAG*G*GTGATATTCACCGGCATCTACACGTTCGAATCAGCTGTAAAAGTGATGGCGCGAGGTTTCATATTACAACCGTTTACTTATCTTAGAGATGCATGGAATTGGTTGGACTTCGTAGTAATAGCATTA*GC*GTGAGTTTTCCAGATACTAATCCTAATTGTGTTCGTTTTTTCTTAATACTCCATATATACAATGCTTTACCCCA

*g*gtgatattcaccggcatctacacgttcgaatcagctgtaaaagtgatggcgcgaggtttc

V I F T G I Y T F E S A V K V M A R G F

atattacaaccgtttacttatcttagagatgcatggaattggttggacttcgtagtaata

I L Q P F T Y L R D A W N W L D F V V I

gcatta*gc*

A L *A*

- **First italicized nucleotide is part of codon spanning exon splice site (see para4 sequence above). Last two italicized nucleotides are part of codon spanning exon splice site (see para6 sequence below).**

**Para6**

**>para6_PR**

GATGGTTGGTGACCGGATTTGATCATCATCGGTTGGATTCAACAG*A*TATGTAACTATGGGTATAGATTTGGGTAATCTCGCTGCATTGAGAACATTCAGGGTACTACGAGCTCTCAAAACAGTGGCCATCGTTCCAGGTGAGTTTCGCCAAATCAATGACAGTCGACTCTTTATAACTTGACGAACTGTGACAAAGTCCAGAACGTATCGCCTAAAACACATCTCTTCTCTTCCCCGCAGGTCTCAAGACCATCGTCGGCGCTGTCATAGAGTCCGTTAAGAATCTCAGAGATGTGATAATTTTAACAATGTTTTCGTTATCGGTGTTTGCTTTAATGGGGCTGCAGATCTACATGGGCGTGCTGACGCAGAAGTGCATCCGGGAGTTCCCGATGGACGGTTCGTGGGGCAACCTGTCGGACGAGAACTGGGAACGGTTCAACAATAACGACTGTACGTTTCGTTCATGGCAAACAAAGCAATTCCGTGGTCCAAGGTATTTGAGATCTAAATGAATGTTTTTCTTTTTTCTTTTACAGCCAATTGGTACTTCTCGGAAACTGGAGACACGCCTCTTTGTGGGAACTCGTCGGGTGCT*GG*GTAGGGCTTTGAATCACAGTTTAACCTTCATTCCGCCAACATA

*a*tatgtaactatgggtatagatttgggtaatctcgctgcattgagaacattcagggtacta

Y V T M G I D L G N L A A L R T F R V L

cgagctctcaaaacagtggccatcgttccag…gtctcaagaccatcgtcggcgctgtcata

R A L K T V A I V P …G L K T I V G A V I

gagtccgttaagaatctcagagatgtgataattttaacaatgttttcgttatcggtgttt

E S V K N L R D V I I L T M F S L S V F

gctttaatggggctgcagatctacatgggcgtgctgacgcagaagtgcatccgggagttc

A L M G L Q I Y M G V L T Q K C I R E F

ccgatggacggttcgtggggcaacctgtcggacgagaactgggaacggttcaacaataac

P M D G S W G N L S D E N W E R F N N N

gact…ccaattggtacttctcggaaactggagacacgcctctttgtgggaactcgtcgggt

D …S N W Y F S E T G D T P L C G N S S G

gct*gg*

A *G*

- **First italicized nucleotide is part of codon spanning exon splice site (see para5 sequence above). Last two italicized nucleotides are part of codon spanning exon splice site (see para7 sequence below). Omitted intronic sequence denoted by “…”**

**>para6_ROCK**

ATGATGGTTGGTGACCGGATTTGATCATCATCGGTTGGATTCAACAG*A*TATGTAACTATGGGTATAGATTTGGGTAATCTCGCTGCATTGAGAACATTCAGGGTACTACGAGCTCTCAAAACAGTGGCCATCGTTCCAGGTGAGTTTCGCCAAATCAATGACAGTAAACTCTTTATAACTTGACTAACTGTGACAAAGTCCAGAACGCATCGCCTAAAACACATCTCTTGTCTTCCCCGCAGGTCTCAAGACCATCGTCGGCGCTGTCATAGAGTCCGTTAAGAATCTCAGAGATGTGATAATTTTAACAATGTTTTCGTTATCGGTGTTTGCTTTAATGGGGCTGCAGATCTACATGGGCGTGCTGACGCAGAAGTGCATCCGGGAGTTCCCGATGGACGGTTCGTGGGGCAACCTGTCGGACGAGAACTGGGAACGGTTCAACAATAACGACTGTGCGTTTCTTTCATGGCGAACAAAGCATTCCACGGTCCAAGATATTTGAGATCTAAATGAATGTTTTTCTTTTTTTCTTTTACAGCCAATTGGTACTTCTCGGAAACTGGAGACACGCCTCTTTGTGGGAACTCGTCGGGTGCT*GG*GTAGGGCTTTGAATCACAGTTTAACCTTCATTCCGCCAACATAC

*a*tatgtaactatgggtatagatttgggtaatctcgctgcattgagaacattcagggtacta

Y V T M G I D L G N L A A L R T F R V L

cgagctctcaaaacagtggccatcgttccag…gtctcaagaccatcgtcggcgctgtcata

R A L K T V A I V P …G L K T I V G A V I

gagtccgttaagaatctcagagatgtgataattttaacaatgttttcgttatcggtgttt

E S V K N L R D V I I L T M F S L S V F

gctttaatggggctgcagatctacatgggcgtgctgacgcagaagtgcatccgggagttc

A L M G L Q I Y M G V L T Q K C I R E F

ccgatggacggttcgtggggcaacctgtcggacgagaactgggaacggttcaacaataac

P M D G S W G N L S D E N W E R F N N N

gact…ccaattggtacttctcggaaactggagacacgcctctttgtgggaactcgtcgggt

D …S N W Y F S E T G D T P L C G N S S G

gct*gg*

A *G*

- **First italicized nucleotide is part of codon spanning exon splice site (see para5 sequence above). Last two italicized nucleotides are part of codon spanning exon splice site (see para7 sequence below). Omitted intronic sequence denoted by “…”**

**Para7**

**>para7_PR**

TTCTCCGCATGTGAAAAAAATATGTAAGCTGGATGAAGGTAAATAACCAGGATTCCAAGATTGTACTAACTTACTGCATCTCTTATTCCTCTTTTTCGTTCGATAG*C*CAATGCGAAGAAGGATATATTTGTTTACAAGGTTATGGAGATAATCCAAATTACGGGTATACAAGTTTCGATACTTTCGGATGGGCATTCTTATCTGCCTTTCGTCTAATGACCCAAGATTATTGGGAGAATCTTTATCAACTGGTTCGTATCCAATGACCGATGTGTGAATGAGACTGAGTATTCATGATCCCTTCCTCCTCAACCTGATTCCTCCAGAACTCCAACAAAAAAATATACCGACCATCATTTATTAAATCTCGATTTGTATCTTTTTTCTTCTGCCCAAACAACTGCAATCAACTTTCAACCACTACAATTATCCCCACTCTCCCCCTACACTTCAAACCAAAACCAACCAACTCTTCGTGGTGTGCAAAACAGGTGTTACGATCAGCTGGACCGTGGCACATGCTCTTCTTCATTGTGATTATCTTCTTGGGTTCGTTCTACCTT**T**TAAATTTGATCTTGGCCATTGTCGCCATGTCGTACGACGAACTCCAGAAGAAGGCCGAAGAGGAAGAGGCCGCCGAGGAAGAAGCGCTTCGGGTGAGCGAATTCTTTTGAATCTGT

*c*caatgcgaagaaggatatatttgtttacaaggttatggagataatccaaattacgggtat

Q C E E G Y I C L Q G Y G D N P N Y G Y

acaagtttcgatactttcggatgggcattcttatctgcctttcgtctaatgacccaagat

T S F D T F G W A F L S A F R L M T Q D

Tattgggagaatctttatcaactg…gtgttacgatcagctggaccgtggcacatgctcttc

Y W E N L Y Q L … V L R S A G P W H M L F

ttcattgtgattatcttcttgggttcgttctaccttttaaatttgatcttggccattgtc

F I V I I F L G S F Y L L N L I L A I V

gccatgtcgtacgacgaactccagaagaaggccgaagaggaagaggccgccgaggaagaa

A M S Y D E L Q K K A E E E E A A E E E

gcgcttcgg

A L R

- **First italicized nucleotide is part of codon spanning exon splice site (see para6 sequence above. Single red nucleotide represents silent mutation found in Puerto Rico sequence. Red, bold and underlined nucleotide represents a G>T mutation corresponding to previously reported V410L *kdr* mutation (Haddi et al. 2017). Mutated amino acid residue is highlighted in red. Omitted intronic sequence denoted by “…”**

**>para7_ROCK**

CAAGATTGTACTAACTTACTGCATCTCTTATTCCTCTTTTTCGTTCGATAG*C*CAATGCGAAGAAGGATATATTTGTTTACAAGGTTATGGAGATAATCCAAATTACGGGTATACAAGTTTCGATACTTTCGGATGGGCATTCTTATCTGCCTTTCGTCTAATGACCCAAGATTATTGGGAGAATCTTTATCAACTGGTTCGTATCCAATGACCGATGTGTGAATGAGACTGAGTATTCATGATCCCTTCCTCCTCAACCTGATTCCTCCAGAACTCCAACAAAAAAATATACCGACCATCATTTATTAAATCTCGATTTGTATCTTTTTTCTTCTGCCCAAACAACTGCAATCAACTTTCAACCACTACAATTATCCCCACTCTCCCCCTACACTTCAAACCAAAACCAACCAACTCTTCGTGGTGTGCAAAACAGGTGTTACGATCAGCTGGACCGTGGCACATGCTCTTCTTCATTGTGATTATCTTCTTGGGTTCGTTCTACCTT**T**TAAATTTGATCTTGGCCATTGTCGCCATGTCGTACGACGAACTCCAGAAGAAGGCCGAAGAGGAAGAGGCCGCCGAGGAAGAAGCGCTTCGGGTGAGCGAATTCTTTTGAATCTGTTTCTTTCTTGAAATCTGTTATGTTTGTTTTTTTGTTTTGGTTTCCCACTCAAATGTATAT

*c*caatgcgaagaaggatatatttgtttacaaggttatggagataatccaaattacgggtat

Q C E E G Y I C L Q G Y G D N P N Y G Y

acaagtttcgatactttcggatgggcattcttatctgcctttcgtctaatgacccaagat

T S F D T F G W A F L S A F R L M T Q D

Tattgggagaatctttatcaactg…gtgttacgatcagctggaccgtggcacatgctcttc

Y W E N L Y Q L … V L R S A G P W H M L F

ttcattgtgattatcttcttgggttcgttctaccttttaaatttgatcttggccattgtc

F I V I I F L G S F Y L L N L I L A I V

gccatgtcgtacgacgaactccagaagaaggccgaagaggaagaggccgccgaggaagaa

A M S Y D E L Q K K A E E E E A A E E E

gcgcttcgg

A L R

- **First italicized nucleotide is part of codon spanning exon splice site (see para6 sequence above). Single red nucleotide represents silent mutation found in Rockefeller sequence. Red, bold and underlined nucleotide represents a G>T mutation corresponding to previously reported V410L *kdr* mutation (Haddi et al. 2017). Mutated amino acid residue is highlighted in red. Omitted intronic sequence denoted by “…”**

**Para8**

**>para8_PR**

CGAAAGTGGCTAAACGTTTTGTTTGACATTAACCGTTGTGCGCCTTCGGCAAAATTTCGCCCAAAACCATCACTAATCGATATCTGATTTACTCCCCCAAAATGCGAACCATCCCCCTACCCCCACCTAACAGGAAGCGGAGGAAGCAGCTGCAGCGAAAGCGGCCAAACTCGAGGCCCAAGCAGCGGCAGCGGCGGCCGCAGCCAACCCGGAGATCGCCAAGAGCCCGTCGGACTTTTCCTGCCACAGCTACGAGCTGTTCGTGAACCAGGAGAAGGGCAACGACGACAACAACAAGGAGAAGATGTCGATCCGGAGCGAAGGATTGGAGTCGGTGAGCGAAATCACAAGAACAACCGCACCAACAGCTACTGCAGCTGGCACTGCAAAAGCCCGTAAAGTGAGCGCGGTAAGTATGGGAACCGCTTTCTGCACCCTCCCCCCACTAGTTCCCTCCCTTTCGTAC

gaagcggaggaagcagctgcagcgaaagcggccaaactcgaggcccaagcagcggcagcg

E A E E A A A A K A A K L E A Q A A A A

gcggccgcagccaacccggagatcgccaagagcccgtcggacttttcctgccacagctac

A A A A N P E I A K S P S D F S C H S Y

gagctgttcgtgaaccaggagaagggcaacgacgacaacaacaaggagaagatgtcgatc

E L F V N Q E K G N D D N N K E K M S I

cggagcgaaggattggagtcggtgagcgaaatcacaagaacaaccgcaccaacagctact

R S E G L E S V S E I T R T T A P T A T

gcagctggcactgcaaaagcccgtaaagtgagcgcg

A A G T A K A R K V S A

- **Silent mutation in Puerto Rico sequence marked in red text.**

**>para8_ROCK**

ATATGTGCTTGTCGAAAGTGGCTAAACGTTTTGCTTGACATTAACCGTTGTGCGCCTTCGGCAAAATTGCGCCCAGTACCCTCACTAATCGATATCTGATTTACTCCCTCAAAATTCGAACCACCCCCACCCCCCTACTCCCACCAAACAGGAAGCGGAGGAAGCAGCTGCAGCGAAAGCGGCCAAACTCGAGGCCCAAGCAGCGGCAGCGGCGGCCGCAGCCAACCCGGAGATCGCCAAGAGCCCGTCGGACTTTTCCTGCCACAGCTACGAGCTGTTCGTGAACCAGGAGAAGGGCAACGACGACAACAACAAGGAGAAGATGTCGATCCGGAGCGAAGGATTGGAGTCGGTGAGCGAAATCACAAGAACAACCGCACCAACAGCTACTGCAGCTGGCACTGCAAAAGCCCGTAAAGTGAGCGCGGTAAGTATGGGAACCGCTTTCTGCACCCTCCCCCCACACTAGTTCCCCCCCTTTCGTACCTTGCCCAT

gaagcggaggaagcagctgcagcgaaagcggccaaactcgaggcccaagcagcggcagcg

E A E E A A A A K A A K L E A Q A A A A

gcggccgcagccaacccggagatcgccaagagcccgtcggacttttcctgccacagctac

A A A A N P E I A K S P S D F S C H S Y

gagctgttcgtgaaccaggagaagggcaacgacgacaacaacaaggagaagatgtcgatc

E L F V N Q E K G N D D N N K E K M S I

cggagcgaaggattggagtcggtgagcgaaatcacaagaacaaccgcaccaacagctact

R S E G L E S V S E I T R T T A P T A T

gcagctggcactgcaaaagcccgtaaagtgagcgcg

A A G T A K A R K V S A

- **Silent mutation in Rockefeller sequence marked in red text.**

**Para9**

**>para9_PR**

ACCGCCAGGGGGTCGCGGCATTCCAGAAGGTAGGTCACTCACTTACATGAATACACTTGAAATTTCCACTAATCCTATATAGAATTAAATGAGATTGAGTTCAAGCAT

ggggtcgcggcattccagaag

G V A A F Q K

**>para9_ROCK**

ACCGCCAGGGGGTCGCGGCATTCCAGAAGGTAGGTCACTCACTTACATGAATACACTTGAAATTTCCACTAATCCTATATAAAATAAATGAGATTGAGTTCAAGCATTGATAAATGAATATAAGAGCAGCGAGGA

ggggtcgcggcattccagaag

G V A A F Q K

**Para10**

**>para10_PR**

CAACAACACACTAACCAATGAAAACCTATACTTACTTTCTAGGCTTCACTTTCATTACCTGGTTCACCATTTAATCTTCGTAGAGGATCTAGAGGATCACATCAGGTACAAGCTTCCCAGACCAAAAACTTCACTAAACGAGTTATACGTGTTTTTCTTCTTCTTCTTTTCTTGCGTTTCGCTCTGTTCTCTTCCCCCAAACACATGCTGCAACGCCACCTGGACACTCCGGACAGTTTACGATACGTAACGGTAGAGGACGTTTCGTGGGCGTACCTGGTAGCGATAGAAAACCATTGGTACTCTCAACATATCTCGATGCACAAGAACATTTGCCATACGCCGATGACTCGAACGCGGTCACACCGATGTCGGAGGAAAATGGTGCAATCATCGTTCCAGTATACTATGCTAATTTA*G*GTACACTATAAAAAAGTAAAATCCAGTTCAATCAAACGACAATCTTCGATTTTCGATTTCGATTCCAACGATCAAAATTTTTCACGATTTGTTGTTACTTCCATCCGTTTCGGAATTCCTTTGCATTTCAACAAAAGAGAAAGATACGATTGCTCTTTCCAAACTGCGATGGCTA

gcttcactttcattacctggttcaccatttaatcttcgtagaggatctagaggatcacat

A S L S L P G S P F N L R R G S R G S H

cag…tttacgatacgtaacggtagaggacgtttcgtgggcgtacctggtagcgatagaaaa

Q … F T I R N G R G R F V G V P G S D R K

ccattggtactctcaacatatctcgatgcacaagaacatttgccatacgccgatgactcg

P L V L S T Y L D A Q E H L P Y A D D S

aacgcggtcacaccgatgtcggaggaaaatggtgcaatcatcgttccagtatactatgct

N A V T P M S E E N G A I I V P V Y Y A

aattta*g*

N L

- **Last italicized nucleotide is part of codon spanning exon splice site (see para11 sequence below).** **Omitted intronic sequence denoted by “…”**

**>para10_ROCK**

CAACAACACACTAACCAATGAAAACCTATACTTACTTTCTAGGCTTCACTTTCATTACCTGGTTCACCATTTAATCTTCGTAGAGGATCTAGAGGATCACATCAGGTACAAGCTTCCCAGATCAAAAACTTCACTAAACGAGTTATACGTGTTTTTCTTCTTCTTCTTTTCTTGCGTTTCGCTCTGTTCTCTTCCCCCAAACACATGCTGCAACGCCACATGGACACTCCGGACAGTTTACGATACGTAACGGTAGAGGACGTTTCGTGGGCGTACCTGGTAGCGATAGAAAACCATTGGTACTCTCAACATATCTCGATGCACAAGAACATTTGCCATACGCCGATGACTCGAACGCGGTCACACCGATGTCGGAGGAAAATGGTGCAATCATCGTTCCAGTATACTATGCTAATTTA*G*GTACACTATAAAAAAGTAAAATCCAGTTCAATCAAACGACAATCTTCGATTTTCGATTTCGATTCCAACGATCAAAATTTCTCACGATTTGTTGTTACTTCCATCCGTTTCGGAATTCCTTTGCATATCAACAAAAGAGAAAGATACGATTGCTCTTTC

gcttcactttcattacctggttcaccatttaatcttcgtagaggatctagaggatcacat

A S L S L P G S P F N L R R G S R G S H

cag…tttacgatacgtaacggtagaggacgtttcgtgggcgtacctggtagcgatagaaaa

Q … F T I R N G R G R F V G V P G S D R K

ccattggtactctcaacatatctcgatgcacaagaacatttgccatacgccgatgactcg

P L V L S T Y L D A Q E H L P Y A D D S

aacgcggtcacaccgatgtcggaggaaaatggtgcaatcatcgttccagtatactatgct

N A V T P M S E E N G A I I V P V Y Y A

aattta*g*

N L

- **Last italicized nucleotide is part of codon spanning exon splice site (see para11 sequence below).** **Omitted intronic sequence denoted by “…”**

**Para11**

**>para11_PR**

AG*GT*TCGCGACATTCATCGTACACATCGCATCAATCGCGCATCTCGTACACATCGCACGGCGACCTGCTCGGCGGCATGACGAAGGAGAGCCGACTGCGGAACCGGTCGGCGCGCAACACCAATCACTCGATCGTGCCGCCGCCGAACATGTCCGGGCCGAACATGTCCTACGTGGACAGCAACCACAAGGGGCAGCGAGACTTTGTGAGTATTACCAAAGATTAATTA**GGGTCGATGTACCAATAGTCGCATAGCTAAGAACAAAAAATCATAGAAAATCGAAAAATAACGCTAGCGTCATTAGTTTTACATCATCTGAAAGCTTTTTATCTTGGTTTTGTGGGAAAAATATGAAAACTACGAAAACTAAAATGTTTGTATTTATTATCGCGTGTGCCACTATAGGAATACATGTGCCTATAGTAGCACTATTTCTAATTCCTGTTCCTATAGTAGCACTAGCATCACGGCGTTGGAAAAGTACTTATCAAAATCGTATTTTTACTAAACTTTTATATTTTTCCTACACAGTGTGGATCAAAAGCTTTCGATTGATGTAAAAAAAGATCTTATTTCATCAATTATTTTGTATAATAAAAAATAGTTTCTCTCAGTAGTGCTACTATAGGAACAATAGGTTAACTATAGGCGCAAAGGAGGTCAAATTTTAAGCAAAACTAATTATTTCGATCATTTTTTGAAAAAAATCAAGCTGTGTATAAATGGGACTTAGATTAATAGCTCCTGACCTTCATGTCAAAAAATATTTTGAAAAGATTTATAGCAAAACGGCCGTAAAATGCCACTAGTACGACTATAGGGGACTGTCCACTAAGGGAACACCGACCC**TATAATAATATATTTTGCATAACCTGTCCATGA

*gt*tcgcgacattcatcgtacacatcgcatcaatcgcgcatctcgtacacatcgcacggcgac

*G* S R H S S Y T S H Q S R I S Y T S H G D

ctgctcggcggcatgacgaaggagagccgactgcggaaccggtcggcgcgcaacaccaat

L L G G M T K E S R L R N R S A R N T N

cactcgatcgtgccgccgccgaacatgtccgggccgaacatgtcctacgtggacagcaac

H S I V P P P N M S G P N M S Y V D S N

cacaaggggcagcgagacttt

H K G Q R D F

- **Bold, underlined text represents intron insertion present in PR but not in ROCK. First two italicized nucleotides are part of codon spanning exon splice site (see para10 sequence above). Red nucleotide in Puerto Rico sequence represents silent mutation.**

**>para11_ROCK**

ATGTACCTTTCGAGCTTATCGATGCATGCGTCCTGGTAGTCGTGGATCCAATTAACACCGTTAAAATGGGCCACACTGCGCATGTCCTCGGGAAGTCGCTCCACCTCGGGCCAATCGAATTGCTGGTCTATGATGGGTATAATGTTGCAGTTCGAGTTCAGGGCAGCCACGATTTCCTGCGAAACGAGTATCCCCTTATGAGTACCTATTGGTTAAAGGTCGATCGGCAAATGCGGTACTCACGCGATGCACCCAGTCCTTGCAGTCCTCGTCGTTAATGCAGCGGTGCAGGGCATCGGGCGTTAATACTAAGACAAAGTTCTTTGCCTGTCGAATACTGTTCAATAGGCCGTTGTCAAATTTGCCCGCACAATGGCTTGCGTTACTAACCGTTAACTGTCCTATTCCACACGATCAATGCTGCTAAAAAAAAG*GT*TCGCGACATTCATCGTACACATCGCATCAATCGCGCATCTCGTACACATCGCACGGCGACCTGCTCGGCGGCATGACGAAGGAGAGCCGACTGCGGAACCGGTCGGCGCGCAACACCAATCACTCGATCGTGCCGCCGCCGAACATGTCCGGGCCGAACATGTCCTACGTGGACAGCAACCACAAGGGGCAGCGAGACTTTGTGAGTATTACCAAAGATTAATTATAATAATATATTTTGCATAACCTGTCCATGAATCAAAGAAAAA

*gt*tcgcgacattcatcgtacacatcgcatcaatcgcgcatctcgtacacatcgcacggcgac

*G* S R H S S Y T S H Q S R I S Y T S H G D

ctgctcggcggcatgacgaaggagagccgactgcggaaccggtcggcgcgcaacaccaat

L L G G M T K E S R L R N R S A R N T N

cactcgatcgtgccgccgccgaacatgtccgggccgaacatgtcctacgtggacagcaac

H S I V P P P N M S G P N M S Y V D S N

cacaaggggcagcgagacttt

H K G Q R D F

- **First two italicized nucleotides are part of codon spanning exon splice site (see para10 sequence above). Red nucleotide in Rockefeller sequence represents silent mutation.**

**Para12**

**>para12_PR**

TAAAAACTGGCTCATTTACTCTTCTACTTTTGTATCAAATTTACATCCAATTCTCTTCTATTTTTACAGGATATGTCACAAGACTGTACAGACGAAGCTGGCAAAATAAAACACAACGACAATCCTTTCATCGAGCCC**A**CTCAAACACAAACCGTAGTAGATATGAAAGGTAAGTGGTCCCTTTCAAATGATCAATGAACCCGATTTGTCTAACCTTCTATCCGCATTTTAGACGTGATGGTGTTAAACGATATCATCGAGCAAGCTGCTGGTCGGCATAGTAGAGCTAGTGATCATGGA*G*GTAATTTGAAGTCACATGTTCTCTTCGGTACTATGAAAAACAAACCGGAAATCAATTCCGTCAAATAGTGCATACTGATGAGAGAAAACGAAAATCACAAACATACATGCATACATACATACATGGTCATTGTCATTACTAGCATAGTCAAATTCATCGAAATCATTTACATCCAACATGACCGACCGTTTCGTTATCGCCCGGATCACGCGAACGTGAAGCCATTTACCCATCGCCATA

gatatgtcacaagactgtacagacgaagctggcaaaataaaacacaacgacaatcctttc

D M S Q D C T D E A G K I K H N D N P F

atcgagcccactcaaacacaaaccgtagtagatatgaaag…acgtgatggtgttaaacgat

I E P T Q T Q T V V D M K …D V M V L N D

atcatcgagcaagctgctggtcggcatagtagagctagtgatcatgga*g*

I I E Q A A G R H S R A S D H G

- **Underlined nucleotide represents a T>A mutation compared to reference & red triplet represents codon that is affected by point mutation – found in exon 20.** **Corresponding amino acid substitution is S722T (Itokawa et al. 2019, Saavedra-Rodriguez et al. 2019). Mutated amino acid residue is highlighted in red.** **Omitted intronic sequence denoted by “…”**

**>para12_ROCK**

TAAAACTGGCTCATTTACTCTTCTACTTTTGTATCAAATTTACATCCAATTCTCTTCTATTTTTACAGGATATGTCACAAGACTGTACAGACGAAGCTGGCAAAATAAAACACAACGACAATCCTTTCATCGAGCCCTCTCAAACACAAACCGTAGTAGATATGAAAGGTAAGTGGTCCCTTTCAAATGATCAATGGACCCGATTTGTCTAACCTTCTATCCGCATTTTAGACGTGATGGTGTTAAACGATATCATCGAGCAAGCTGCTGGTCGGCATAGTAGAGCTAGTGATCATGGA*G*GTAATTTGAAGTCACATGTTCTCTTCGGTACTATGAAAAACAAACCGGAAATCAATTCCGTCAAATAGTGCATACTGATGAGAGAAAACGAAAATCACATACATACATGCATACATACATACATGGTCATTGTCATTACTAGCATAGTCAAATTCATCGAAATCATTTACATCCAACATGACCGACCGTTTCGTTATCGCCCGGATCACGCGAACGTGAAGCCATTTACCCATCGCCATA

gatatgtcacaagactgtacagacgaagctggcaaaataaaacacaacgacaatcctttc

D M S Q D C T D E A G K I K H N D N P F

atcgagccctctcaaacacaaaccgtagtagatatgaaag…acgtgatggtgttaaacgat

I E P S Q T Q T V V D M K …D V M V L N D

atcatcgagcaagctgctggtcggcatagtagagctagtgatcatgga*g*

I I E Q A A G R H S R A S D H G

- **Omitted intronic sequence denoted by “…”**

**Para13-1**

**>para13-1_PR**

TCTTCATGGTCGACCGGCTGTGTGGCTCTCTGTTTTCAGTCTCTGTTTACTACTTCCCCACAGAGGACGACGACGAGGACGGTCCAACGTTCAAGGACAAGGCCCTGGAGTTCACGATGCGGATGATCGACGTCTTCTGCGTGTGGGACTGCTGCTGGGTGTGGCTCAAGTTCCAGGAGTGGGTTGCCTTCATTGTGTTCGACCCGTTCGTCGAGCTGTTCATCACCCTGTGTATCGTGGTCAACACGCTGTTCATGGCCCTGGATCACCACGATATGGACCCGGACATGGAGCGGGCCCTCAAGAGTGGTAACTATGTGAGTAGCGCACCAGGCCTGGGGGATCCTCCCAAGCCCCGGAACAGAGGGGGTGATCCAACGAATTTAACCTTGACTGTGTGTCTTCTATATTTCGATTCCTTAGTTTTTCACGGCGACCTTCGCGATAGAAGCAACGATGAAGCTGATTGCGATGAGTCCCAA

gtctctgtttactacttccccacagaggacgacgacgaggacggtccaacgttcaaggac

V S V Y Y F P T E D D D E D G P T F K D

aaggccctggagttcacgatgcggatgatcgacgtcttctgcgtgtgggactgctgctgg

K A L E F T M R M I D V F C V W D C C W

gtgtggctcaagttccaggagtgggttgccttcattgtgttcgacccgttcgtcgagctg

V W L K F Q E W V A F I V F D P F V E L

ttcatcaccctgtgtatcgtggtcaacacgctgttcatggccctggatcaccacgatatg

F I T L C I V V N T L F M A L D H H D M

gacccggacatggagcgggccctcaagagtggtaactat…tttttcacggcgaccttcgcg

D P D M E R A L K S G N Y … F F T A T F A

atagaagcaacgatgaagctgattgcgatgagtccc

I E A T M K L I A M S P

- **Red nucleotide in Puerto Rico sequence represents silent mutation. Omitted intronic sequence denoted by “…”**

**>para13-1_ROCK**

TTCATGGTCGACCGGCTGTGTGGCTCTCTGTTTTCAGTCTCTGTTTACTACTTCCCCACAGAGGACGACGACGAGGACGGTCCAACGTTCAAGGACAAGGCCCTGGAGTTCACGATGCGGATGATCGACGTCTTCTGCGTGTGGGACTGCTGCTGGGTGTGGCTTAAGTTCCAGGAGTGGGTTGCCTTCATTGTGTTCGACCCGTTCGTCGAGCTGTTCATCACCCTGTGTATCGTGGTCAACACGCTGTTCATGGCCCTGGATCACCACGATATGGACCCGGACATGGAGCGGGCCCTCAAGAGTGGTAACTATGTGAGTAGCGCACCAGGCCTGGGGGATCCTCCCAAGCCCCGGAACAGAGGGGGTGATCCAACGAATTTAACCTTGACTGTGTGTCTTCTATATTTCGATTCTTTAGTTTTTCACGGCGACCTTCGCGATAGAAGCAACGATGAAGCTGATTGCGATGAGTCCCA

gtctctgtttactacttccccacagaggacgacgacgaggacggtccaacgttcaaggac

V S V Y Y F P T E D D D E D G P T F K D

aaggccctggagttcacgatgcggatgatcgacgtcttctgcgtgtgggactgctgctgg

K A L E F T M R M I D V F C V W D C C W

gtgtggcttaagttccaggagtgggttgccttcattgtgttcgacccgttcgtcgagctg

V W L K F Q E W V A F I V F D P F V E L

ttcatcaccctgtgtatcgtggtcaacacgctgttcatggccctggatcaccacgatatg

F I T L C I V V N T L F M A L D H H D M

gacccggacatggagcgggccctcaagagtggtaactat…tttttcacggcgaccttcgcg

D P D M E R A L K S G N Y … F F T A T F A

atagaagcaacgatgaagctgattgcgatgagtccc

I E A T M K L I A M S P

- **Omitted intronic sequence denoted by “…”**

**Para13-2**

**>para13-2_PR**

CTGTGTATCGTGGTCAACACGCTGTTCATGGCCCTGGATCACCACGATATGGACCCGGACATGGAGCGGGCCCTCAAGAGTGGTAACTATGTGAGTAGCGCACCAGGCCTGGGGGATCCTCCCAAGCCCCGGAACAGAGGGGGTGATCCAACGAATTTAACCTTGACTGTGTGTCTTCTATATTTCGATTCCTTAGTTTTTCACGGCGACCTTCGCGATAGAAGCAACGATGAAGCTGATTGCGATGAGTCCCAAGTACTACTTCCAAGAGGGCTGGAACATATTCGATTTCATCATCGTGGCGCTGTCGTTGCTCGAGCTGGGTCTGGAAGGTGTTCAGGGATTGTCAGTATTACGTTCATTCCGTTTGGTAAGTATGGTTCGGAAGAGGGTGTACGAGGGATCCTCGTTTGTCCTTGGTTATTTAGGTTAGTACTCCGTGTCTAGTGGTTCCCG

ctgtgtatcgtggtcaacacgctgttcatggccctggatcaccacgatatggacccggac

L C I V V N T L F M A L D H H D M D P D

atggagcgggccctcaagagtggtaactat…tttttcacggcgaccttcgcgatagaagca

M E R A L K S G N Y … F F T A T F A I E A

acgatgaagctgattgcgatgagtcccaagtactacttccaagagggctggaacatattc

T M K L I A M S P K Y Y F Q E G W N I F

gatttcatcatcgtggcgctgtcgttgctcgagctgggtctggaaggtgttcagggattg

D F I I V A L S L L E L G L E G V Q G L

tcagtattacgttcattccgtttg

S V L R S F R L

- **Silent mutation in Puerto Rico sequence marked in red text. Omitted intronic sequence denoted by “…”**

**>para13-2_ROCK**

CCTGTGTATCGTGGTCAACACGCTGTTCATGGCCCTGGATCACCACGATATGGACCCGGACATGGAGCGGGCCCTCAAGAGTGGTAACTATGTGAGTAGCGCACCAGGCCTGGGGGATCCTCCCAAGCCCCGGAACAGAGGGGGTGATCCAACGAATTTAACCTTGACTGTGTGTCTTCTATATTTCGATTCTTTAGTTTTTCACGGCGACCTTCGCGATAGAAGCAACGATGAAGCTGATTGCGATGAGTCCCAAGTACTACTTCCAAGAGGGCTGGAACATATTCGATTTCATCATCGTGGCGCTGTCGCTGCTCGAGCTGGGTCTGGAAGGTGTTCAGGGATTGTCAGTATTACGTTCATTCCGTTTGGTAAGTATGGTTCGGAAAAGGGTGTACGAGGGATCCTCGTTTGTCCTTGGTTATTTAGGTTAGTACTCCGTGTCTAGTGGTTCCC

cctgtgtatcgtggtcaacacgctgttcatggccctggatcaccacgatatggacccggac

L C I V V N T L F M A L D H H D M D P D

atggagcgggccctcaagagtggtaactat…tttttcacggcgaccttcgcgatagaagca

M E R A L K S G N Y … F F T A T F A I E A

acgatgaagctgattgcgatgagtcccaagtactacttccaagagggctggaacatattc

T M K L I A M S P K Y Y F Q E G W N I F

gatttcatcatcgtggcgctgtcgctgctcgagctgggtctggaaggtgttcagggattg

D F I I V A L S L L E L G L E G V Q G L

tcagtattacgttcattccgtttg

S V L R S F R L

- **Omitted intronic sequence denoted by “…”**

**Para 14**

**>para14_PR**

CAGCTAAGAGTGTTCAAACTAGCGAAATCGTGGCCAACGCTAAACTTGCTGATATCGATCATGGGCCGCACGGTGGGCGCTCTCGGTAATCTGACCTTCGTCCTGTGTATCATCATCTTCATCTTCGCTGTGATGGGAATGCAACTGTTCGGCAAGAACTACACAGGTTAGCGTTCGTCGGGGAGCAGCTGTGCGGCCTGTTTGCCTCCTATATAG

ctaagagtgttcaaactagcgaaatcgtggccaacgctaaacttgctgatatcgatcatg

L R V F K L A K S W P T L N L L I S I M

ggccgcacggtgggcgctctcggtaatctgaccttcgtcctgtgtatcatcatcttcatc

G R T V G A L G N L T F V L C I I I F I

ttcgctgtgatgggaatgcaactgttcggcaagaactacaca

F A V M G M Q L F G K N Y T

- **Red nucleotides represent silent mutations in Puerto Rico.**

**>para14_ROCK**

AGGGGAAGACAATCAGGACACTTCAGGGGGAGTCCATCTGGACACTCCAGGCCTGGAAGTCTGGAAACTCCATGGGAGTCCAGTTGAAATTCCCGGTATTCTCTGCTGAAATTTTCGTACTCCTACAACTACGTTTTAGGACTGTAATAGTTAGAAAGAGTATGTGAAACGTCTGTACGTCGAATTATCACATGTCTACTAGAGCGTTTTCAAATACTATCAGCAAAAACCCTGAAAATACTAACACATGCGCGTTCTCTACACAGCTAAGAGTGTTCAAACTAGCGAAATCGTGGCCAACGCTAAACTTGCTGATATCGATCATGGGCCGCACGGTGGGCGCTCTCGGTAATCTGACCTTCGTCCTGTGTATCATCATCTTCATCTTCGCTGTGATGGGAATGCAACTGTTCGGCAAGAACTACACAGGTTAGCGTTTCGTCGGGGAGCAGCTGTGCGGCCTGTTTTGCCTCCTATATAGTTCACATTTTTTCGAACTACTATTTTCTTCTTCCACCAGA

ctaagagtgttcaaactagcgaaatcgtggccaacgctaaacttgctgatatcgatcatg

L R V F K L A K S W P T L N L L I S I M

ggccgcacggtgggcgctctcggtaatctgaccttcgtcctgtgtatcatcatcttcatc

G R T V G A L G N L T F V L C I I I F I

ttcgctgtgatgggaatgcaactgttcggcaagaactacaca

F A V M G M Q L F G K N Y T

- **Red nucleotides represent silent mutations in Rockefeller.**

**Para15**

**>para15_PR**

CACTCAACACCAAAAAAAATACCATAATTTCGCGCCAAAAAACCTCACACAGCTTCGAGTGTTCAAGCTAGCGAAATCGTGGCCGACGTTGAACTTACTCATTTCCATCATGGGTCGAACGATGGGTGCGTTAGGTAATCTGACGTTTGTGCTCTGCATTATCATCTTCATCTTTGCCGTGATGGGAATGCAGCTGTTCGGCAAGAACTACATCGGTAAGTATTCGGAAAACGACGAAACATCGAGAGATTCATTTAGGCTAAGTTTTTAAGTTTAACGAACTTTCATCAACGACTGCGAAAAGGGGCGCGAATTTCGAAATACAGTTTCACTCAAGACTTCCATAGACACAAAACAAGCTACGAAGAACTTGTACATAGTTTTCCAGAACAGCATTTCGAATGGAAGCTTACTTTAGATGACGTAGTCAGAAAACTTCTATTTTGATCGGACTCTAGA

cttcgagtgttcaagctagcgaaatcgtggccgacgttgaacttactcatttccatcatg

L R V F K L A K S W P T L N L L I S I M

ggtcgaacg**atg**ggtgcgttaggtaatctgacgtttgtgctctgcattatcatcttcatc

G R T **M** G A L G N L T F V L C I I I F I

tttgccgtgatgggaatgcagctgttcggcaagaactacatc

F A V M G M Q L F G K N Y I

- **This exon and previous exon represent alternate splice variants. Bold, underlined M represents different amino acid residue than in previous exon.**

**>para15_ROCK**

CAACACCAAAAAAAAATACCATAATTTCGCGCCAAAAAACCTCACACAGCTTCGAGTGTTCAAGCTAGCGAAATCGTGGCCGACGTTGAACTTACTCATTTCCATCATGGGTCGAACGATGGGTGCGTTAGGTAATCTGACGTTTGTGCTCTGCATTATCATCTTCATCTTTGCCGTGATGGGAATGCAGCTGTTCGGCAAGAACTACATCGGTAAGTATTCGAAAACGATGAAACATCGAGAGATTCATTTAGGCTAAGTTTATAAGTTTAACGAACTACCATCAACGACTGCGAAAAGGGGCGCGAATTTCAAAATACAGTTCCATTCAAGACTTCCATAGACACAAAACAAGCTACGAAGAACGTGTACATAGTTTTCCAGAACAGCATTTCGAATGGAAGCTTACTTTAGATGACGTAGTCAGAAAACTTCTATTTTGATCGGACTCTA

cttcgagtgttcaagctagcgaaatcgtggccgacgttgaacttactcatttccatcatg

L R V F K L A K S W P T L N L L I S I M

ggtcgaacg**atg**ggtgcgttaggtaatctgacgtttgtgctctgcattatcatcttcatc

G R T **M** G A L G N L T F V L C I I I F I

tttgccgtgatgggaatgcagctgttcggcaagaactacatcg

F A V M G M Q L F G K N Y I

- **This exon and previous exon represent alternate splice variants. Bold, underlined M represents different amino acid residue than in previous exon.**

**Para16-1**

**>para16-1_PR**

ACTCATCTCCACTAGCTCTTAACTAGTATTTCTAATCACCTTATGCTAAGACTTCACTTTATCCTCTATAATCTCGTATAAAATACTGAACAAACGTTTCTAAAACCATCCCCCCAGACAATGTGGATCGCTTCCCGGACAAAGACCTGCCACGGTGGAACTTCACCGACTTCATGCACTCATTCATGATCGTGTTCCGGGTATTATGCGGCGAGTGGATCGAATCCATGTGGGATTGTATGCTTGTGGGTGACGTGTCCTGTATTCCGTTCTTTTTGGCCACCGTAGTGATAGGAAATCTAGTAGTAAGTATTCCGTTTGGGAGTTCTTCTATAAGGCTGACTGAAAGTAAATTGGAGCGCACAACAAGACCTGTTATGCTGTAAGTTCCAGCACTAAATTTCTCAGGTTGAATTGCAGTAGTTCAATCGAAATCTCGAACTTTCATTTTGATAACAGCAATACTAGACGCGCATAGAACATACAAATTTACATATAGTCAGCCTTTCATGCATTCTATCGTGCTAACCGACAAATTGTTTCCCACCCGCACAG**ATA**CTTAACCTTTTCTTA

gacaatgtggatcgcttcccggacaaagacctgccacggtggaacttcaccgacttcatgcac

D N V D R F P D K D L P R W N F T D F M H

tcattcatgatcgtgttccgggtattatgcggcgagtggatcgaatccatgtgggattgt

S F M I V F R V L C G E W I E S M W D C

atgcttgtgggtgacgtgtcctgtattccgttctttttggccaccgtagtgataggaaat

M L V G D V S C I P F F L A T V V I G N

ctagta…atacttaaccttttctta

L V … I L N L F L

- **Red nucleotide represents silent mutation found in Puerto Rico sequence. Red, bold and underlined nucleotide represents a G>A mutation corresponding to previously reported V1016I *kdr* mutation (Saavedra-Rodriguez et al. 2007). Mutated amino acid residue is highlighted in red. Omitted intron sequence denoted as “…”**

**>para16-1_ROCK**

ACTCATCTCCACTAGCTCTTAACTAGTATTTCTAATCACCTTATGCTAAGACTTCACTTTAACCTCTATAATCTCGTATAAAATACTGAACAAACGTTTCTAAAACCCTCCCCCCAGACAATGTGGATCGCTTCCCGGACAAGGACCTGCCACGGTGGAACTTCACCGACTTTATGCACTCATTCATGATCGTGTTCCGGGTATTATGCGGCGAGTGGATCGAATCCATGTGGGATTGTATGCTTGTGGGTGACGTGTCCTGTATTCCGTTCTTTTTGGCCACCGTAGTGATAGGAAATCTAGTAGTAAGTATTCCGTTTGGAAGTTCATCTGTAAGGCTGACTGAAAGTAAATTGGAGCGCACAACAGACCTATTATGCTGTAATTCGTGATTCAACTAGTTAAAAAAGACCGTTGATCTTGATAGCATCAACACTAAAGGCGTGCTAGCAGCGAGCGAGGGGCGTACCAATTTACTTTTAGTCAGCCTTTCTTGCATTCTATCGTGCTAACCGACAAATTGTTTCCCACCCGCACAGGTACTTAACCTTTTCTTAGCCTTGCTTTTGTCCAATTTCGGTTCATCCTCGCTGTCGGCACCGACGGCCGACAACGA

gacaatgtggatcgcttcccggacaaggacctgccacggtggaacttcaccgactttatg

D N V D R F P D K D L P R W N F T D F M

cactcattcatgatcgtgttccgggtattatgcggcgagtggatcgaatccatgtgggat

H S F M I V F R V L C G E W I E S M W D

tgtatgcttgtgggtgacgtgtcctgtattccgttctttttggccaccgtagtgatagga

C M L V G D V S C I P F F L A T V V I G

aatctagta…gtacttaaccttttcttagccttgcttttgtccaatttcggttcatcctcg

N L V … V L N L F L A L L L S N F G S S S

ctgtcggcaccgacggccgacaac

L S A P T A D N

- **Red nucleotide represents silent mutation found in Rockefeller sequence. Omitted intron sequence denoted as “…”**

**Para16-2**

**>para16-2_PR**

TACTTTTGGTCAGCCTTTCTTGCATTCTATCGTGCTAACCGACAAATTGTTTCCCACCCGCACAG**A**TACTTAACCTTTTCTTAGCCTTGCTTTTGTCCAATTTCGGTTCATCCTCGCTGTCGGCACCGACGGCCGACAACGAAACGAACAAGATCGCGGAGGCGTTCAATCGGATATCGCGCTTCTCCAACTGGATCAAGTCGAACATCGCCAACGCGCTCAAGTTCGTGAAAAACAAGTTAACAAGCCAGATTGCGTCCGTGCAGCCCGCAGGTGAGCAGCACAATCATCTCAGTTGGATATGGAACGAA*G*GTTATTATCCACTTGCGTTTTCTAACACTAAACTAAATAACTATCTGTTACTACTACTACCGCTACTAAACGAAACTTAACCCGTTGTTGAGACCCCATGCCCTTAAATCGTCGCCATTGTGTTTCCCCCGTGTAATATCCCACATTCCCGCTAGAAACTCAA

atacttaaccttttcttagccttgcttttgtccaatttcggttcatcctcgctgtcggca

I L N L F L A L L L S N F G S S S L S A

ccgacggccgacaacgaaacgaacaagatcgcggaggcgttcaatcggatatcgcgcttc

P T A D N E T N K I A E A F N R I S R F

tccaactggatcaagtcgaacatcgccaacgcgctcaagttcgtgaaaaacaagttaaca

S N W I K S N I A N A L K F V K N K L T

agccagattgcgtccgtgcagcccgcaggtgagcagcacaatcatctcagttggatatgg

S Q I A S V Q P A G E Q H N H L S W I W

aacgaa*g*

N E

- **Red, bold and underlined nucleotide represents a G>A mutation corresponding to previously reported V1016I *kdr* mutation (Saavedra-Rodriguez et al. 2007). Mutated amino acid residue is highlighted in red. Last italicized nucleotide is part of codon spanning exon splice site (see para17 sequence below).**

**>para16-2_ROCK**

CCCGCACAGGTACTTAACCTTTTCTTAGCCTTGCTTTTGTCCAATTTCGGTTCATCCTCGCTGTCGGCACCGACGGCCGACAACGAAACGAACAAGATCGCGGAGGCGTTCAATCGGATATCGCGCTTCTCCAACTGGATCAAGTCGAACATCGCCAACGCACTCAAGTTCGTGAAAAACAAGTTAACAAGCCAGATTGCGTCCGTGCAGCCCGCAGGTGAGCAGCACAATCATCTCAGTTGGATATGGAACGAA*G*GTTATTATCCACTTGCGTTTTCTAACACTAAACTAAATAACTATCTGTTACTACTACTACCGCTACTAAAC

gtacttaaccttttcttagccttgcttttgtccaatttcggttcatcctcgctgtcggca

V L N L F L A L L L S N F G S S S L S A

ccgacggccgacaacgaaacgaacaagatcgcggaggcgttcaatcggatatcgcgcttc

P T A D N E T N K I A E A F N R I S R F

tccaactggatcaagtcgaacatcgccaacgcactcaagttcgtgaaaaacaagttaaca

S N W I K S N I A N A L K F V K N K L T

agccagattgcgtccgtgcagcccgcaggtgagcagcacaatcatctcagttggatatgg

S Q I A S V Q P A G E Q H N H L S W I W

aacgaa*g*

N E

- **Last italicized nucleotide is part of codon spanning exon splice site (see para17 sequence below). Red nucleotide in Rockefeller sequence represents silent mutation.**

**Para17**

**>para17_PR**

CACACAGAACGATGCGATACACAATTCGAACATGTTGATACTGAACTCGTTACCCTGTTCTTTTTTGGCTATCGTCTTTTTTCTTCCAAATACGTTAAACCGTACGTCCTCCTTCACGATTTGGATCGCAAAACAAAAACCATTTCGATCAAAATTGGAACTCTTCAAATTACGTAAAATCCCATTTTGAAACCATGAAATAAAAAAAAATCAAATCCATGATCTGCGGGGACCGCACGGAAAAACACGATAG*GC*AAAGGGGTATGTCCATGTATCTCTGCAGAGCATGGTGAAAATGAGCTGGAATTAACTCCAGATGACATACTGGCCGACGGGCTGCTCAAGAAAGGCGTCAAGGAGCACAACCAGCTGGAGGTGGCCATCGGCGACGGGATGGAGTTCACGATACACGGCGACCTGAAGAACAAGGGCAAGAAGAACAAACAGCTGATGAACAATTCCAAGGTAATTGGCTCCTCTGGTGGGGCGCGATGCACATGTTCATTACACACACATTTCTTTCAATTCACCAAACATCTCTCATTTGCGACTTCAC

*gc*aaaggggtatgtccatgtatctctgcagagcatggtgaaaatgagctggaattaactcca

*G* K G V C P C I S A E H G E N E L E L T P

gatgacatactggccgacgggctgctcaagaaaggcgtcaaggagcacaaccagctggag

D D I L A D G L L K K G V K E H N Q L E

gtggccatcggcgacgggatggagttcacgatacacggcgacctgaagaacaagggcaag

V A I G D G M E F T I H G D L K N K G K

aagaacaaacagctgatgaacaattccaag

K N K Q L M N N S K

- **First two italicized nucleotides are part of codon spanning exon splice site (see para16-2 sequence above).**

**>para17_ROCK**

AAACCGTACGTCCTCCTTCACGATTTGGACCGCAAAACAAAAACCATTTCGATCAAAATTGGAACTTTTCAAATTACGTAAAATCCCATTTTGAAATCATGAAATCAAAAAAAAAATCAAATCCATGATCTGCGGGGACCGCACGGAAAAACACGATAG*GC*AAAGGGGTATGTCCATGTATCTCTGCAGAGCATGGTGAAAATGAGCTGGAATTAACTCCAGATGACATACTGGCCGACGGGCTGCTCAAGAAAGGCGTCAAAGAGCACAACCAGCTGGAGGTGGCCATCGGCGACGGGATGGAGTTCACGATACACGGCGACCTGAAGAACAAGGGCAAGAAGAACAAACAGCTGATGAACAATTCCAAGGTAATTGGCTCCTCTGGTGGGGCGCGATGCACATGTTCATTACACACAC

*gc*aaaggggtatgtccatgtatctctgcagagcatggtgaaaatgagctggaattaactcca

*G* K G V C P C I S A E H G E N E L E L T P

gatgacatactggccgacgggctgctcaagaaaggcgtcaaagagcacaaccagctggag

D D I L A D G L L K K G V K E H N Q L E

gtggccatcggcgacgggatggagttcacgatacacggcgacctgaagaacaagggcaag

V A I G D G M E F T I H G D L K N K G K

aagaacaaacagctgatgaacaattccaag

K N K Q L M N N S K

- **First two italicized nucleotides are part of codon spanning exon splice site (see para16-2 sequence above). Red nucleotide in Rockefeller sequence represents silent mutation.**

**Para18-1**

**>para18-1_PR**

TATATCTTGATAATTAGGTGATAGGCAATTCTATTAGTAATCATCAAGATAATAAATTAGAGCACGAACTGAATCATAGAGGCATGTCCTTGCAGGACGATGATACTGCCAGTATAAAGTCCTATGGCAGTCACAAGAATCGCCCCTTCAAGGACGAAAGCCACAAAGGCAGCGCCGAAACGATGGAGGGCGAAGAAAAGCGTGACGTCAGCAAGGAGGACCTAGGAATTGATGAAGGTAAGGGCCGTACATGAATTCTTCAGGGAATTTCTTTTTTGACATTAATTTCATTTGTTGCGCAGAGCTAGACGATGAGTGCGATGGCGAGGAAGGTCCACTGGATGGCGAACTGATCATCCACGCCGACGAGGACGAAGTGATAGAGGACTCACCAGCGGACTGTTGTCCGGACAATTGCTACAAAAAGTTTCCGGTCCTGGCAGGGGACGACGATGCGCCATTCTGGCAGGGCTGGGCCAATCTGCGACTGAAAACGTTTCAGCTCATCGAGAACAAGTACTTTGAGACGGCCGTCATCACGATGATTCTGCTGAGTAGTTTGGCCCTGGTGAGTACGATGTTGAAGTGTGGCACATAACAATCTGAGCACCGTTGTGGCACTTTGTCGAACACTGAACTAATGGACGGTTCCGGTTTCAGGCTCTCGAGGATGTGCATCTTCCCCATCGACCAATCCTGCAGGACGTCCTGTACTACATGGACAGGATATTCACGGTGATATTTTTTCTAGAGATGTTAATCAAGTGGTTGGCGCTGGGTTTTCGAGTTTACTTTACGAACGCCTGGTGCTGGCTCGATTTCATAATTGTCATGGTAAGTA

gtgataggcaattctattagtaatcatcaagataataaattagagcacgaactgaatcat

V I G N S I S N H Q D N K L E H E L N H

agaggcatgtccttgcaggacgatgatactgccagtataaagtcctatggcagtcacaag

R G M S L Q D D D T A S I K S Y G S H K

aatcgccccttcaaggacgaaagccacaaaggcagcgccgaaacgatggagggcgaagaa

N R P F K D E S H K G S A E T M E G E E

aagcgtgacgtcagcaaggaggacctaggaattgatgaag…agctagacgatgagtgcgat

K R D V S K E D L G I D E …E L D D E C D

ggcgaggaaggtccactggatggcgaactgatcatccacgccgacgaggacgaagtgata

G E E G P L D G E L I I H A D E D E V I

gaggactcaccagcggactgttgtccggacaattgctacaaaaagtttccggtcctggca

E D S P A D C C P D N C Y K K F P V L A

ggggacgacgatgcgccattctggcagggctgggccaatctgcgactgaaaacgtttcag

G D D D A P F W Q G W A N L R L K T F Q

ctcatcgagaacaagtactttgagacggccgtcatcacgatgattctgctgagtagtttg

L I E N K Y F E T A V I T M I L L S S L

gccctg…gctctcgaggatgtgcatcttccccatcgaccaatcctgcaggacgtcctgtac

A L … A L E D V H L P H R P I L Q D V L Y

tacatggacaggatattcacggtgatattttttctagagatgttaatcaagtggttggcg

Y M D R I F T V I F F L E M L I K W L A

ctgggttttcgagtttactttacgaacgcctggtgctggctcgatttcataattgtcatg

L G F R V Y F T N A W C W L D F I I V M

- **Red nucleotides represent silent mutations found in Puerto Rico sequence. Omitted intron sequence represented by “…”**

**>para18-1_ROCK**

GTTCTATGTTGTTATATCTTGATAATTAGGTGATAGGCAATTCTATTAGTAATCATCAAGATAATAAATTAGAGCACGAACTGAATCATAGAGGCATGTCCTTGCAGGACGATGATACTGCCAGTATAAAGTCCTATGGCAGTCACAAGAATCGCCCCTTCAAGGACGAAAGCCACAAGGGCAGCGCCGAAACGATGGAGGGCGAAGAAAAGCGTGACGTCAGCAAGGAGGACCTAGGAATTGATGAAGGTAAGAAGAATCGCCGCCAACGAATTGTTCAGGGGACCCTTGTTTAACGCTTTTTTGCGCCTTATTTATTCCGCAGAGCTAGATGATGAGTGCGATGGCGAGGAAGGTCCACTGGATGGCGAACTGATCATCCACGCCGACGAGGACGAAGTGATAGAGGACTCACCAGCGGACTGTTGTCCGGACAATTGCTACAAAAAGTTTCCGGTCCTGGCAGGGGACGACGATGCGCCATTCTGGCAGGGCTGGGCCAATCTGCGACTGAAAACGTTTCAGCTCATCGAGAACAAGTACTTTGAGACGGCCGTCATCACGATGATTCTGCTGAGTAGTTTGGCCCTGGTGAGTACGATGTTGAAGTGTGGCACATAACGATCTGAGCACCGTTGTGCCACTTTTTCAAACAGTGAATTAACGGACGGTTCCGGTTTCAGGCTCTCGAGGATGTGCATCTTCCCCATCGACCAATCCTGCAGGACGTCCTGTACTACATGGACAGGATATTCACGGTGATATTTTTTCTAGAGATGTTAATCAAGTGGTTGGCGCTGGGTTTTCGAGTTTACTTTACGAACGCCTGGTGCTGGCTCGATTTCATAATTGTCATGGTAAGTAGCCCCCAACTAAAG

gtgataggcaattctattagtaatcatcaagataataaattagagcacgaactgaatcat

V I G N S I S N H Q D N K L E H E L N H

agaggcatgtccttgcaggacgatgatactgccagtataaagtcctatggcagtcacaag

R G M S L Q D D D T A S I K S Y G S H K

aatcgccccttcaaggacgaaagccacaagggcagcgccgaaacgatggagggcgaagaa

N R P F K D E S H K G S A E T M E G E E

aagcgtgacgtcagcaaggaggacctaggaattgatgaag…agctagatgatgagtgcgat

K R D V S K E D L G I D E …E L D D E C D

ggcgaggaaggtccactggatggcgaactgatcatccacgccgacgaggacgaagtgata

G E E G P L D G E L I I H A D E D E V I

gaggactcaccagcggactgttgtccggacaattgctacaaaaagtttccggtcctggca

E D S P A D C C P D N C Y K K F P V L A

ggggacgacgatgcgccattctggcagggctgggccaatctgcgactgaaaacgtttcag

G D D D A P F W Q G W A N L R L K T F Q

ctcatcgagaacaagtactttgagacggccgtcatcacgatgattctgctgagtagtttg

L I E N K Y F E T A V I T M I L L S S L

gccctg…gctctcgaggatgtgcatcttccccatcgaccaatcctgcaggacgtcctgtac

A L … A L E D V H L P H R P I L Q D V L Y

tacatggacaggatattcacggtgatattttttctagagatgttaatcaagtggttggcg

Y M D R I F T V I F F L E M L I K W L A

ctgggttttcgagtttactttacgaacgcctggtgctggctcgatttcataattgtcatg

L G F R V Y F T N A W C W L D F I I V M

- **Red nucleotides represent silent point mutations in Rockefeller sequence. Omitted intron sequence represented by “…”**

**Para18-2**

**>para18-2_PR**

AGGACGAAGTGATAGAGGACTCACCAGCGGACTGTTGTCCGGACAATTGCTACAAAAAGTTTCCGGTCCTGGCAGGGGACGACGATGCGCCATTCTGGCAGGGCTGGGCCAATCTGCGACTGAAAACGTTTCAGCTCATCGAGAACAAGTACTTTGAGACGGCCGTCATCACGATGATTCTGCTGAGTAGTTTGGCCCTGGTGAGTACGATGTTGAAGTGTGGCACATAACAATCTGAGCACCGTTGTGGCACTTTGTCGAACACTGAACTAATGGACGGTTCCGGTTTCAGGCTCTCGAGGATGTGCATCTTCCCCATCGACCAATCCTGCAGGACGTCCTGTACTACATGGACAGGATATTCACGGTGATATTTTTTCTAGAGATGTTAATCAAGTGGTTGGCGCTGGGTTTTCGAGTTTACTTTACGAACGCCTGGTGCTGGCTCGATTTCATAATTGTCATGGTAAGTAGCCCCCCACTAAAGTGTGTTCCCCTTTCGATCTCTATCTCTTGAGCCCCTAAAGA

aggacgaagtgatagaggactcaccagcggactgttgtccggacaattgctacaaaaagttt

D E V I E D S P A D C C P D N C Y K K F

ccggtcctggcaggggacgacgatgcgccattctggcagggctgggccaatctgcgactg

P V L A G D D D A P F W Q G W A N L R L

aaaacgtttcagctcatcgagaacaagtactttgagacggccgtcatcacgatgattctg

K T F Q L I E N K Y F E T A V I T M I L

ctgagtagtttggccctg…gctctcgaggatgtgcatcttccccatcgaccaatcctgcag

L S S L A L … A L E D V H L P H R P I L Q

gacgtcctgtactacatggacaggatattcacggtgatattttttctagagatgttaatc

D V L Y Y M D R I F T V I F F L E M L I

aagtggttggcgctgggttttcgagtttactttacgaacgcctggtgctggctcgatttc

K W L A L G F R V Y F T N A W C W L D F

ataattgtcatg

I I V M

- **Omitted intron sequence represented by “…”**

**>para18-2_ROCK**

GTGATAGAGGACTCACCAGCGGACTGTTGTCCGGACAATTGCTACAAAAAGTTTCCGGTCCTGGCAGGGGACGACGATGCGCCATTCTGGCAGGGCTGGGCCAATCTGCGACTGAAAACGTTTCAGCTCATCGAGAACAAGTACTTTGAGACGGCCGTCATCACGATGATTCTGCTGAGTAGTTTGGCCCTGGTGAGTACGATGTTGAAGTGTGGCACATAACGATCTGAGCACCGTTGTGCCACTTTTTCAAACAGTGAATTAACGGACGGTTCCGGTTTCAGGCTCTCGAGGATGTGCATCTTCCCCATCGACCAATCCTGCAGGACGTCCTGTACTACATGGACAGGATATTCACGGTGATATTTTTTCTAGAGATGTTAATCAAGTGGTTGGCGCTGGGTTTTCGAGTTTACTTTACGAACGCCTGGTGCTGGCTCGATTTCATAATTGTCATGGTAAGTAGCCCCCAACTAAAGTGTGTTCCCCTTTCGATCTCTATCTCTTGA

gtgatagaggactcaccagcggactgttgtccggacaattgctacaaaaagtttccggtc

V I E D S P A D C C P D N C Y K K F P V

ctggcaggggacgacgatgcgccattctggcagggctgggccaatctgcgactgaaaacg

L A G D D D A P F W Q G W A N L R L K T

tttcagctcatcgagaacaagtactttgagacggccgtcatcacgatgattctgctgagt

F Q L I E N K Y F E T A V I T M I L L S

agtttggccctg…gctctcgaggatgtgcatcttccccatcgaccaatcctgcaggacgtc

S L A L … A L E D V H L P H R P I L Q D V

ctgtactacatggacaggatattcacggtgatattttttctagagatgttaatcaagtgg

L Y Y M D R I F T V I F F L E M L I K W

ttggcgctgggttttcgagtttactttacgaacgcctggtgctggctcgatttcataatt

L A L G F R V Y F T N A W C W L D F I I

gtcatg

V M

- **Omitted intron sequence represented by “…”**

**Para19**

**>para19_PR**

GCGCCGACCCAGATTGCGGTCAGGTTAATGAGCGACAGCTGATGATGGCGGGTTGGTGAATTTTGGTGACGTGAACGATGGGAAAATTAAGTATCGATTTGGAGAATCGGATTAGTAAAAATAAAAGAAAAATGTAAAAATAACCCATGGGACAATATTGTTTGATACAAATTTGCACATAATTGAGTAGTTTATCGAGAATTTTGTTAGAATTTAATTAAACTTGGCTATCATCAACAGTTGTCATGCCAATTTTAATCAAATTCTAACAAAATTCTCGATAAACTACTCAATTATGTGCAAATTTGTATCAAACAATATTGTCCCATGGGTTATTTTTACATTTTTCTTTTATTTTTACTAATCCGATTCTCCAAATCGATACTTAATTTTCCCATCGTTCACGTCACCAAAATTCACCAACCCGCCATCATCAGCTGTCGCTCATTAACCTGACCGCAATCTGGGTCGGCGCCGCGGACATTCCGGCGTTCCGCTCGATGCGTACCCTGAGGGCGCTGCGCCCACTGCGAGCCGTTTCTCGCTGGGAGGGTATGAGAGTAAGTAGTTCCATCAACGCACAGACACACGCACACCCAATCGCATCGGCAGGCAATGCCGGCTCACAAGTCACCGAGCAAGACGGAGCCGCGGCCATTGGATCCGATCTTGCCGGGGTTGT

ctgtcgctcattaacctgaccgcaatctgggtcggcgccgcggacattccggcgttccgc

L S L I N L T A I W V G A A D I P A F R

tcgatgcgtaccctgagggcgctgcgcccactgcgagccgtttctcgctgggagggtatg

S M R T L R A L R P L R A V S R W E G M

aga

R

**>para19_ROCK**

GCTATCATCAACAGTTGTCATGCCAATTTTAATCAAATTTTAACAAAACACTCAATTATGTGCAAATTTGTATCAAACAATATTGTCCCATGGATTATTTTTAGATTTTATTTTTATTTTTACTAATCCGATTCTCCAAATCGATACTTAATTTTCCCATCGTACACGTCACCAAAATTCACCAACCCGCCATCATCAGCTGTCGCTCATTAACCTGACCGCAATCTGGGTCGGCGCCGCGGACATTCCGGCGTTCCGCTCGATGCGTACCCTGAGGGCGCTGCGCCCACTGCGAGCCGTTTCTCGCTGGGAGGGTATGAGAGTAAGTAGTTCCATCAACGCACAGACACACGCACACTCAATCGCATCGGCAGGCAATGCCGGCTCACAAGTCACCGAGCAAGATGGAGCCGCGGCCATTGGATCCGATCTTGCCGGGGTT

ctgtcgctcattaacctgaccgcaatctgggtcggcgccgcggacattccggcgttccgc

L S L I N L T A I W V G A A D I P A F R

tcgatgcgtaccctgagggcgctgcgcccactgcgagccgtttctcgctgggagggtatg

S M R T L R A L R P L R A V S R W E G M

aga

R

**Para20**

**>para20_PR**

CGTGTGTGTTACTCGTTTGTTTTACCCGAAATCTCTAGGTGTCCTTAATCAACTTCGTTGCTTCACTCTGTGGAGCTGGTGGTATTCAAGCATTCAAAACAATGCGAACTCTTAGAGCACTGAGACCGCTACGTGCCATGTCCCGTATGCAGGGTATGAGGGTACGTAGACTCCAAACCAAAATCTAATTCTTTCGTTGAACCGAACCGAACTTCATGCTCAAAATTGAAAGTTCTCATCATATTCCAAATCCAAATGCTCTTCAGTTCCAATCGATCGATTCTCACACATTTTATAATTCGCTCTCTAATTTCTGAATTTCGATTTTCTACTCTTTCTTCCCCGA

gtgtccttaatcaacttcgttgcttcactctgtggagctggtggtattcaagcattcaaa

V S L I N F V A S L C G A G G I Q A F K

acaatgcgaactcttagagcactgagaccgctacgtgccatgtcccgtatgcagggtatg

T M R T L R A L R P L R A M S R M Q G M

agg

R

**>para20_ROCK**

TCCTCACTCTCGGTGTGTTACTCGTTTGTTTTACCCGAAATCTCTAGGTGTCCTTAATCAACTTCGTTGCTTCACTCTGTGGAGCTGGTGGTATTCAAGCATTCAAAACAATGCGAACTCTTAGAGCACTGAGACCGCTACGTGCCATGTCCCGTATGCAGGGTATGAGGGTACGTAGACTCCAAACCAAAATCTAATTCTTTCGTTGAACCGAACCGAACTTCATGCTCAAAATTGAAAGTTCTCATCATATTCCAAATCCAAATGCTCTTCAGTTCCAATCGATCGATTCTCACACATTTTATAATTCGCTCTCTAATTTCTGAATTACGATTTTCTAC

gtgtccttaatcaacttcgttgcttcactctgtggagctggtggtattcaagcattcaaa

V S L I N F V A S L C G A G G I Q A F K

acaatgcgaactcttagagcactgagaccgctacgtgccatgtcccgtatgcagggtatg

T M R T L R A L R P L R A M S R M Q G M

agg

R

**Para21-1**

**>para21-1_PR**

**T**AAAGGCTAGTGATATTCAAATGAAAATTAATGCTAATTAATCATTTCCCAACTCTCTATTCCCGCTTGGATCGTCCTTCTTTTATTTAGGTTGTCGTCAATGCATTGGTACAGGCTATACCGTCCATCTTCAACGTGTTATTGGTGTGTTTGATCTTTTGGTTGATTTTCGCTATTATGGGTGTGCAGCTGTTTGCTGGCAAGTATTTTAAGGTGAGTGTACACTTCTAAAGTTGGATCCAAAAGGGCTTGATGAATAACCATCGGATGTTGCCTCCCTTCAGTGCGTCGACAAGAACAAGACGACGCTGTCGCACGAGATCATTCCGGATGTGAACGCGTGCGTCGCGGAGAACTACACGTGGGAGAACTCGCCGATGAACTTCGACCACGTGGGGAAGGCGTACCTGTGTCTGTTCCAGGTGGCAACGTTCAAGGGCTGGATCCAGATCATGAACGACGCCATCGACTCGCGGGAGGTAAGTTATTGTGAAATCGAACTTGTTACGAATGATCTGCTTACAATTTTACGTCCTCGATCCTTCCAGGTGGGAAAGCAGCCGATTCGCGAGACCAACATCTACATGTACCTCTACTTTGTGTTCTTCATCATCT**G**CGGGTCGTTCTTCACGCTGAATCTGTTCATCGGTGTCATCATCGACAACT

gttgtcgtcaatgcattggtacaggctataccgtccatcttcaacgtgttattggtgtgt

V V V N A L V Q A I P S I F N V L L V C

ttgatcttttggttgattttcgctattatgggtgtgcagctgtttgctggcaagtatttt

L I F W L I F A I M G V Q L F A G K Y F

aag…tgcgtcgacaagaacaagacgacgctgtcgcacgagatcattccggatgtgaacgcg

K … C V D K N K T T L S H E I I P D V N A

tgcgtcgcggagaactacacgtgggagaactcgccgatgaacttcgaccacgtggggaag

C V A E N Y T W E N S P M N F D H V G K

gcgtacctgtgtctgttccaggtggcaacgttcaagggctggatccagatcatgaacgac

A Y L C L F Q V A T F K G W I Q I M N D

gccatcgactcgcgggag…gtgggaaagcagccgattcgcgagaccaacatctacatgtac

A I D S R E … V G K Q P I R E T N I Y M Y

ctctactttgtgttcttcatcatctgcgggtcgttcttcacgctgaatctgttcatcggt

L Y F V F F I I C G S F F T L N L F I G

gtcatcatcgacaac

V I I D N

- **Red, bold and underlined nucleotide represents a T>G mutation corresponding to previously reported F1534 *kdr* mutation (Kawada et al. 2009). Mutated amino acid residue is highlighted in red. Omitted intronic sequence denoted by “…”**

**>para21-1_ROCK**

GTTAAAGGCTATTGATATTCAAATGAAAATTAATCATTTCCCAACTCTCTATTACCGCTTGGATCGTCCTTCCTTTATTTAGGTTGTCGTCAATGCATTGGTACAGGCTATACCGTCCATCTTCAACGTGTTATTGGTGTGTTTGATCTTTTGGTTGATTTTCGCTATTATGGGTGTGCAGCTGTTTGCTGGCAAGTATTTTAAGGTGAGTATACACTTCTAAAGTTGGATCCAAAAGGGCTTGATGAATAACCATCGGATGTTGCCTCCCTGCAGTGCGTCGATAAGAACAAGACGACGCTGTCGCACGAGATCATTCCGGATGTGAACGCGTGCGTCGCGGAGAACTACACGTGGGAGAACTCGCCGATGAACTTCGACCACGTGGGGAAGGCGTACCTGTGTCTGTTCCAGGTGGCAACGTTCAAGGGCTGGATCCAGATCATGAACGACGCCATCGACTCGCGGGAGGTAAGTTATTGTGAAATCGAACTTGTTGCGAATGATCTGCTTACAATTTTGCGTCCTCGATCCTTCCAGGTGGGAAAGCAGCCGATTCGCGAGACCAACATCTACATGTACCTCTACTTCGTGTTCTTCATCATCTTCGGGTCGTTCTTCACGCTGAATCTGTTCATCGGTGTCATCATCGACAACTT

gttgtcgtcaatgcattggtacaggctataccgtccatcttcaacgtgttattggtgtgt

V V V N A L V Q A I P S I F N V L L V C

ttgatcttttggttgattttcgctattatgggtgtgcagctgtttgctggcaagtatttt

L I F W L I F A I M G V Q L F A G K Y F

aag…tgcgtcgataagaacaagacgacgctgtcgcacgagatcattccggatgtgaacgcg

K … C V D K N K T T L S H E I I P D V N A

tgcgtcgcggagaactacacgtgggagaactcgccgatgaacttcgaccacgtggggaag

C V A E N Y T W E N S P M N F D H V G K

gcgtacctgtgtctgttccaggtggcaacgttcaagggctggatccagatcatgaacgac

A Y L C L F Q V A T F K G W I Q I M N D

gccatcgactcgcgggag…gtgggaaagcagccgattcgcgagaccaacatctacatgtac

A I D S R E … V G K Q P I R E T N I Y M Y

ctctacttcgtgttcttcatcatcttcgggtcgttcttcacgctgaatctgttcatcggt

L Y F V F F I I F G S F F T L N L F I G

gtcatcatcgacaactt

V I I D N

- **Silent mutations in Rockefeller sequence denoted by red text. Omitted intronic sequence denoted by “…”**

**Para21-2**

**>para21-2_PR**

GCAGCCGATTCGCGAGACCAACATCTACATGTACCTCTACTTTGTGTTCTTCATCATCT**G**CGGGTCGTTCTTCACGCTGAATCTGTTCATCGGTGTCATCATCGACAACTTCAACGAGCAGAAGAAGAAAGCCGGTGGCTCACTGGAAATGTTCATGACGGAGGATCAGAAAAAGTACTACAACGCCATGAAAAAGATGGGCTCGAAGAAGCCGCTGAAAGCTATTCCACGGCCTAGGGTAAGGCATTTCCATCGCACATCAACTGTGACGTATTCCTTCCTAATCTCGCTATTCTCAATTTCAGTGGCGACCACAAGCAATAGTATTCGAAATAGTTACCAATAAGAAGTTCGACATGATCATCATGTTGTTCATCGGGTTCAACATGTTGACGATGACGCTCGATCACTACAAGCAGACGGACACGTTTAGCGCGGTGCTAGACTATCTGAACATGATCTTCATCTGCATCTTCAGTAGCGAGTGTCTGATGAAGATTTTCGCGCTGCGGTATCACTACTTTATCGAGCCGTGGAACCTGTTCGATTTCGTCGTCGTCATCCTGTCCATTTTA*G*GTGTGTAAAGATATGAGTTTCGCCTTTTATATTGATGGGATTTTTTCGAATAAGATGTTAAGCGAAAGTATCGTTAAGAACGCAGTTTGCGTGGCTTAAGACTGTGCTTTTTGACCACAGCTTCTTCTGA

gcagccgattcgcgagaccaacatctacatgtacctctactttgtgttcttcatcatctgc

Q P I R E T N I Y M Y L Y F V F F I I C

gggtcgttcttcacgctgaatctgttcatcggtgtcatcatcgacaacttcaacgagcag

G S F F T L N L F I G V I I D N F N E Q

aagaagaaagccggtggctcactggaaatgttcatgacggaggatcagaaaaagtactac

K K K A G G S L E M F M T E D Q K K Y Y

aacgccatgaaaaagatgggctcgaagaagccgctgaaagctattccacggcctagg…tgg

N A M K K M G S K K P L K A I P R P R … W

cgaccacaagcaatagtattcgaaatagttaccaataagaagttcgacatgatcatcatg

R P Q A I V F E I V T N K K F D M I I M

ttgttcatcgggttcaacatgttgacgatgacgctcgatcactacaagcagacggacacg

L F I G F N M L T M T L D H Y K Q T D T

tttagcgcggtgctagactatctgaacatgatcttcatctgcatcttcagtagcgagtgt

F S A V L D Y L N M I F I C I F S S E C

ctgatgaagattttcgcgctgcggtatcactactttatcgagccgtggaacctgttcgat

L M K I F A L R Y H Y F I E P W N L F D

ttcgtcgtcgtcatcctgtccatttta*g*

F V V V I L S I L

- **Red, bold and underlined nucleotide represents a T>G mutation corresponding to previously reported F1534 *kdr* mutation (Kawada et al. 2009). Mutated amino acid residue is highlighted in red. Last italicized nucleotide is part of codon spanning exon splice site (see para22-1 sequence below). Omitted intronic sequence denoted by “…”**

**>para21-2_ROCK**

CGCTGAATCTGTTCATCGGTGTCATCATCGACAACTTTAACGAGCAGAAGAAGAAAGCCGGTGGCTCACTGGAAATGTTCATGACGGAGGATCAGAAAAAGTACTACAACGCCATGAAAAAGATGGGCTCGAAGAAGCCGCTGAAAGCTATTCCACGGCCTAGGGTAAGGCATTTCCATCGCACATCAACTGTGACGTATTCCTTCCTAATCTCGCTATTCTCAATTTCAGTGGCGACCACAAGCAATAGTATTCGAAATAGTTACCAATAAGAAGTTCGACATGATCATCATGTTGTTCATCGGGTTCAACATGTTGACGATGACGCTCGATCACTACAAGCAGACGGACACGTTTAGCGCGGTGCTAGACTATCTGAACATGATCTTCATCTGCATCTTCAGTAGCGAGTGTCTGATGAAGATTTTCGCGCTGCGGTATCACTACTTTATCGAGCCGTGGAACCTGTTCGATTTCGTCGTCGTCATCCTGTCCATTTTA*G*

cgctgaatctgttcatcggtgtcatcatcgacaactttaacgagcagaagaagaaagccggt

L N L F I G V I I D N F N E Q K K K A G

ggctcactggaaatgttcatgacggaggatcagaaaaagtactacaacgccatgaaaaag

G S L E M F M T E D Q K K Y Y N A M K K

Atgggctcgaagaagccgctgaaagctattccacggcctagg…tggcgaccacaagcaata

M G S K K P L K A I P R P R … W R P Q A I

gtattcgaaatagttaccaataagaagttcgacatgatcatcatgttgttcatcgggttc

V F E I V T N K K F D M I I M L F I G F

aacatgttgacgatgacgctcgatcactacaagcagacggacacgtttagcgcggtgcta

N M L T M T L D H Y K Q T D T F S A V L

gactatctgaacatgatcttcatctgcatcttcagtagcgagtgtctgatgaagattttc

D Y L N M I F I C I F S S E C L M K I F

gcgctgcggtatcactactttatcgagccgtggaacctgttcgatttcgtcgtcgtcatc

A L R Y H Y F I E P W N L F D F V V V I

ctgtccatttta*g*

L S I L

- **Silent mutation found in Rockefeller sequence marked in red text – this mutation appears to be heterologous in sequence, with both T & C present at this position. Last italicized nucleotide is part of codon spanning exon splice site (see para22-1 sequence below). Omitted intronic sequence denoted by “…”**

**Para22-1**

**>para22-1_PR**

GACCGATGAACCCTGAACTTACCAATAGTCATTCTGAATTCTTCCTCCTGGCCAACCAGAGCATTTAGACCTCTTATGAAAACTTTACCAGAAAACAGGACAAGACATGTATGAATGCTCCCAAAGCGGAAACATAATTCAAATTAATCAATCACTTACCTTAATTAGTCAAATTTTAATTTAATCTATGAGTAAAACTTGCTCAAACACTAAATTCACGTTACTCTCATTCCCTCCCTCGCAG*GT*CTCGTTCTTAGCGATCTCATCGAGAAGTACTTCGTGTCGCCCACGTTGCTCCGAGTCGTCCGAGTGGCCAAGGTCGGTCGTGTGCTGCGTCTCGTCAAGGGTGCCAAAGGTATCCGAACGTTGCTGTTTGCGCTGGCCATGTCCCTGCCGGCGCTGTTCAACATCTGTCTGCTGCTGTTCTTGGTCATGTTCATCTTCGCCATCTTCGGCATGTCGTTCTTCATGCACGTGAAGGACAAGAGCGGGCTGGACGATGTGTACAATTTCAAGACGTTCGGCCAGAGCATGATCCTGCTGTTTCAGGTGAGTTGATGATCGGTATAATGATGAGGGAAGGTGACTGATTTGGTTTGCTTTTTGTTTGCGCAGATGTCTACATCGGCCGGTTGGGATGGCGTGTTGGACGGTATCATCAACGA

*gt*ctcgttcttagcgatctcatcgagaagtacttcgtgtcgcccacgttgctccgagtcgtc

*G* L V L S D L I E K Y F V S P T L L R V V

cgagtggccaaggtcggtcgtgtgctgcgtctcgtcaagggtgccaaaggtatccgaacg

R V A K V G R V L R L V K G A K G I R T

ttgctgtttgcgctggccatgtccctgccggcgctgttcaacatctgtctgctgctgttc

L L F A L A M S L P A L F N I C L L L F

ttggtcatgttcatcttcgccatcttcggcatgtcgttcttcatgcacgtgaaggacaag

L V M F I F A I F G M S F F M H V K D K

agcgggctggacgatgtgtacaatttcaagacgttcggccagagcatgatcctgctgttt

S G L D D V Y N F K T F G Q S M I L L F

cag…atgtctacatcggccggttgggatggcgtgttggacggtatcatcaac

Q … M S T S A G W D G V L D G I I N

- **First two italicized nucleotides are part of codon spanning exon splice site (see para21-2 sequence above). Omitted intronic sequence denoted by “…”**

**>para22-1_ROCK**

TGACCGATGAACCCTGAACTTACCAATAGTCATTCTGAACTCTTCCTCCTGGCCAACCAGAGCATTTAGACCTCTTATGAAAACTTTACCAGAAGACAGGACAGGACATGTATGAATGCTCCCAAAGCGGAAAAATAATTCAAATGAATCAATCACTTACCCTAATTAGTCAAATTTTAATTTAATCTATGAGTAAAACTTGCTCAAACACTAAATTCACATTACTCTCATTCCCTCCCTCGCAG*GT*CTCGTTCTTAGCGATCTCATCGAGAAGTACTTCGTGTCGCCCACGTTGCTCCGAGTCGTTCGAGTGGCCAAGGTCGGTCGTGTGCTGCGTCTCGTCAAGGGCGCCAAAGGTATCCGAACGTTGCTGTTTGCGCTGGCCATGTCCCTGCCGGCGCTGTTCAACATCTGTCTGCTGCTGTTCTTGGTCATGTTCATCTTCGCCATCTTCGGCATGTCGTTCTTCATGCACGTGAAGGACAAGAGCGGGCTGGACGATGTGTACAATTTCAAGACGTTCGGCCAGAGCATGATCCTGCTGTTTCAGGTGAGTTGATGATCGGTATAATGATGAGGGAAGGTGACTGATTTGGTTTGCTTTTTGCTTGCGCAGATGTCTACGTCGGCCGGTTGGGATGGCGTGTTGGACGGTATCATCAACG

*gt*ctcgttcttagcgatctcatcgagaagtacttcgtgtcgcccacgttgctccgagtcgtt

*G* L V L S D L I E K Y F V S P T L L R V V

cgagtggccaaggtcggtcgtgtgctgcgtctcgtcaaggg**c**gccaaaggtatccgaacg

R V A K V G R V L R L V K G A K G I R T

ttgctgtttgcgctggccatgtccctgccggcgctgttcaacatctgtctgctgctgttc

L L F A L A M S L P A L F N I C L L L F

ttggtcatgttcatcttcgccatcttcggcatgtcgttcttcatgcacgtgaaggacaag

L V M F I F A I F G M S F F M H V K D K

agcgggctggacgatgtgtacaatttcaagacgttcggccagagcatgatcctgctgttt

S G L D D V Y N F K T F G Q S M I L L F

cag…atgtctacgtcggccggttgggatggcgtgttggacggtatcatcaac

Q … M S T S A G W D G V L D G I I N

- **First two italicized nucleotides are part of codon spanning exon splice site (see para21-2 sequence above). Red nucleotides represent silent point mutations in Rockefeller sequence. Omitted intronic sequence denoted by “…”**

**Para22-2**

**>para22-2_PR**

GACTGATTTGGTTTGCTTTTTGTTTGCGCAGATGTCTACATCGGCCGGTTGGGATGGCGTGTTGGACGGTATCATCAACGAGGACGAATGCCTGCCGCCGGATAACGATAAGGGTTATCCTGGGAACTGCGGGTCGGCAACGATCGGCATCACGTATTTGCTAGCATATCTTGTTATTAGTTTTTTGATCGTTATCAACATGTACATCGCTGTCATTCTCGAAAATTACTCGCAAGCCACGGAGGACGTGCAGGAGGGTCTAACGGACGACGACTACGATATGTACTATGAGATCTGGCAGCAGTTTGATCCGGACGGTACGCAGTACATCCGGTACGATCAATTGTCAGACTTTTTGGACGTGCTAGAACCGCCACTGCAGATTCATAAGCCGAACAAATACAAGATCATTTCGATGGACATACCGATCTGTCGAGGCGACATGATGTTCTGTGTGGACATTCTAGATGCGTTGACGAAGGACTTCTTCGCTCGGAAAGGTAATCCGATCGAGGAAACGGCCGAACTGGGTGAAGTTCAGGCACGGCCGGATGAAGTGGG

atgtctacatcggccggttgggatggcgtgttggacggtatcatcaacgaggacgaatgc

M S T S A G W D G V L D G I I N E D E C

ctgccgccggataacgataagggttatcctgggaactgcgggtcggcaacgatcggcatc

L P P D N D K G Y P G N C G S A T I G I

acgtatttgctagcatatcttgttattagttttttgatcgttatcaacatgtacatcgct

T Y L L A Y L V I S F L I V I N M Y I A

gtcattctcgaaaattactcgcaagccacggaggacgtgcaggagggtctaacggacgac

V I L E N Y S Q A T E D V Q E G L T D D

gactacgatatgtactatgagatctggcagcagtttgatccggacggtacgcagtacatc

D Y D M Y Y E I W Q Q F D P D G T Q Y I

cggtacgatcaattgtcagactttttggacgtgctagaaccgccactgcagattcataag

R Y D Q L S D F L D V L E P P L Q I H K

ccgaacaaatacaagatcatttcgatggacataccgatctgtcgaggcgacatgatgttc

P N K Y K I I S M D I P I C R G D M M F

tgtgtggacattctagatgcgttgacgaaggacttcttcgctcggaaaggtaatccgatc

C V D I L D A L T K D F F A R K G N P I

gaggaaacggccgaactgggtgaagttcaggcacggccggatgaagtg

E E T A E L G E V Q A R P D E V

**>para22-2_ROCK**

TGAGGGAAGGTGACTGATTTGGTTTGCTTTTTGCTTGCGCAGATGTCTACGTCGGCCGGTTGGGATGGCGTGTTGGACGGTATCATCAACGAGGACGAATGCCTGCCGCCGGATAACGATAAGGGTTATCCTGGGAACTGCGGGTCGGCAACGATCGGCATCACGTATTTGCTAGCATATCTTGTTATTAGTTTTTTGATCGTTATCAACATGTACATCGCTGTCATTCTCGAAAATTACTCGCAAGCCACGGAGGACGTGCAGGAGGGTCTAACGGACGACGACTACGATATGTACTATGAGATCTGGCAGCAGTTTGATCCGGACGGTACGCAGTACATCCGGTACGATCAATTGTCGGACTTTTTGGACGTGCTAGAACCGCCACTGCAGATTCATAAGCCGAACAAATACAAGATCATTTCGATGGACATACCGATCTGTCGAGGCGACATGATGTTCTGTGTGGACATTCTAGATGCGTTGACGAAGGACTTCTTCGCTCGGAAAGGTAATCCGATCGAGGAAACGGCTGAACTGGGTGAAGTTCAGGCACGGCCGGATGAAGTGGGGTATGAACCA

atgtctacgtcggccggttgggatggcgtgttggacggtatcatcaacgaggacgaatgc

M S T S A G W D G V L D G I I N E D E C

ctgccgccggataacgataagggttatcctgggaactgcgggtcggcaacgatcggcatc

L P P D N D K G Y P G N C G S A T I G I

acgtatttgctagcatatcttgttattagttttttgatcgttatcaacatgtacatcgct

T Y L L A Y L V I S F L I V I N M Y I A

gtcattctcgaaaattactcgcaagccacggaggacgtgcaggagggtctaacggacgac

V I L E N Y S Q A T E D V Q E G L T D D

gactacgatatgtactatgagatctggcagcagtttgatccggacggtacgcagtacatc

D Y D M Y Y E I W Q Q F D P D G T Q Y I

cggtacgatcaattgtcggactttttggacgtgctagaaccgccactgcagattcataag

R Y D Q L S D F L D V L E P P L Q I H K

ccgaacaaatacaagatcatttcgatggacataccgatctgtcgaggcgacatgatgttc

P N K Y K I I S M D I P I C R G D M M F

tgtgtggacattctagatgcgttgacgaaggacttcttcgctcggaaaggtaatccgatc

C V D I L D A L T K D F F A R K G N P I

gaggaaacggctgaactgggtgaagttcaggcacggccggatgaagtggggtatgaacca

E E T A E L G E V Q A R P D E V G Y E P

- **Red nucleotides represent silent point mutations in Rockefeller sequence.**

**Para22-3**

**>para22-3_PR**

TCGCTCGGAAAGGTAATCCGATCGAGGAAACGGCCGAACTGGGTGAAGTTCAGGCACGGCCGGATGAAGTGGGGTATGAACCAGTATCTTCAACGCTGTGGCGCCAGCGGGAAGAGTATTGCGCCCGAGTGATACAACACGCGTGGCGGAAGCACAAAGAGCGCCAAGCAGGCGGAGGTGGCGGTGACGACACCGACGCCGATGCCTGTGATAACGACGACGGTGACGACGGTGGTGGTGGTGCTGGTGATGGTGGTAGTGCCGGCGGAGGCGGAGTTACTAGTCCAGGCGTAGGTAGTGGTAGTATCGTCGGCGGAGGAACAACGCCGGGCTCTGGCGGCGGCGGTTCTCAGGCTAACTTAGGTATAGTAGTGGAGCACAATCTATCACCAAAGGAATCGCCCGATGGCAATAATGATCCTCAAGGCCGTCAAACGGCCGTCCTAGTAGAAAGTGATGGATTTGTAACTAAAAACGGTCACCGTGTCGTGATACACTCACGATCGCCGAGTATAACTTCACGATCGGCGGATGTCTGAGCCAGGCCTCGCCCCCCTCTCTCTGATTCAGATTCAGAAGCACGACAGAAATAATATTTAAGAGAAACATAAGAGTACTCTAAACAAACAAAACCAACAAAAAGTTAAAAACAAAAAAAAACTATTCA

tcgctcggaaaggtaatccgatcgaggaaacggccgaactgggtgaagttcaggcacggccg

A R K G N P I E E T A E L G E V Q A R P

gatgaagtggggtatgaaccagtatcttcaacgctgtggcgccagcgggaagagtattgc

D E V G Y E P V S S T L W R Q R E E Y C

gcccgagtgatacaacacgcgtggcggaagcacaaagagcgccaagcaggcggaggtggc

A R V I Q H A W R K H K E R Q A G G G G

ggtgacgacaccgacgccgatgcctgtgataacgacgacggtgacgacggtggtggtggt

G D D T D A D A C D N D D G D D G G G G

gctggtgatggtggtagtgccggcggaggcggagttactagtccaggcgtaggtagtggt

A G D G G S A G G G G V T S P G V G S G

agtatcgtcggcggaggaacaacgccgggctctggcggcggcggttctcaggctaactta

S I V G G G T T P G S G G G G S Q A N L

ggtatagtagtggagcacaatctatcaccaaaggaatcgcccgatggcaataatgatcct

G I V V E H N L S P K E S P D G N N D P

caaggccgtcaaacggccgtcctagtagaaagtgatggatttgtaactaaaaacggtcac

Q G R Q T A V L V E S D G F V T K N G H

cgtgtcgtgatacactcacgatcgccgagtataacttcacgatcggcggatgtctga

R V V I H S R S P S I T S R S A D V -

**>para22-3_ROCK**

AAGGTAATCCGATCGAGGAAACGGCTGAACTGGGTGAAGTTCAGGCACGGCCGGATGAAGTGGGGTATGAACCAGTATCTTCAACGCTGTGGCGCCAGCGGGAAGAGTATTGCGCCCGAGTGATACAACACGCGTGGCGGAAGCACAAAGAGCGCCAAGCAGGCGGAGGTGGCGGTGACGACACCGACGCCGATGCCTGTGATAACGACGACGGTGACGATGGTGGTGGTGGTGCTGGTGATGGTGGTAGTGCCGGCGGAGGCGGAGTTACTAGTCCAGGTGTAGGTAGTGGTAGTATCGTCGGTGGAGGAACAACGCCGGGCTCTGGCGGCGGCGGTTCTCAGGCTAACTTAGGTATAGTAGTGGAGCACAATCTATCACCAAAGGAATCGCCCGATGGCAATAATGATCCTCAAGGCCGTCAAACGGCCGTCCTAGTAGAAAGTGATGGATTTGTAACTAAAAACGGTCACCGTGTCGTGATACACTCACGATCGCCGAGTATAACTTCACGATCGGCGGATGTCTGAGCCAGGCCTCGCCCCCCTCTCTCTGATTCAGATTCAGAAGCACGACAGAAATAATATTTAAGAGAAACATAAGAGTACTCTAAACAAACAAAACCA

aaggtaatccgatcgaggaaacggctgaactgggtgaagttcaggcacggccggatgaagtg

G N P I E E T A E L G E V Q A R P D E V

gggtatgaaccagtatcttcaacgctgtggcgccagcgggaagagtattgcgcccgagtg

G Y E P V S S T L W R Q R E E Y C A R V

atacaacacgcgtggcggaagcacaaagagcgccaagcaggcggaggtggcggtgacgac

I Q H A W R K H K E R Q A G G G G G D D

accgacgccgatgcctgtgataacgacgacggtgacgatggtggtggtggtgctggtgat

T D A D A C D N D D G D D G G G G A G D

ggtggtagtgccggcggaggcggagttactagtccaggtgtaggtagtggtagtatcgtc

G G S A G G G G V T S P G V G S G S I V

ggtggaggaacaacgccgggctctggcggcggcggttctcaggctaacttaggtatagta

G G G T T P G S G G G G S Q A N L G I V

gtggagcacaatctatcaccaaaggaatcgcccgatggcaataatgatcctcaaggccgt

V E H N L S P K E S P D G N N D P Q G R

caaacggccgtcctagtagaaagtgatggatttgtaactaaaaacggtcaccgtgtcgtg

Q T A V L V E S D G F V T K N G H R V V

atacactcacgatcgccgagtataacttcacgatcggcggatgtctga

I H S R S P S I T S R S A D V -

- **Red nucleotides represent silent point mutations in Rockefeller sequence.**

**References**

**Haddi, K., H. V. V. Tome, Y. Du, W. R. Valbon, Y. Nomura, G. F. Martins, K. Dong, and E. E. Oliveira. 2017.** Detection of a new pyrethroid resistance mutation (V410L) in the sodium channel of *Aedes aegypti*: a potential challenge for mosquito control. *Sci. Rep.* 7: 46549.

**Itokawa, K., T. Sekizuka, Y. Maekawa, K. Yatsu, O. Komagata, M. Sugiura, T. Sasaki, T. Tomita, M. Kuroda, K. Sawabe, and S. Kasai. 2019.** High-throughput genotyping of a full voltage-gated sodium channel gene via genomic DNA using target capture sequencing and analytical pipeline MoNaS to discover novel insecticide resistance mutations. *PLoS Negl. Trop. Dis.* 13: e0007818.

**Kawada, H., Y. Higa, O. Komagata, S. Kasai, T. Tomita, N. Thi Yen, L. L. Loan, R. A. Sanchez, and M. Takagi. 2009.** Widespread distribution of a newly found point mutation in voltage-gated sodium channel in pyrethroid-resistant *Aedes aegypti* populations in Vietnam. *PLoS Negl. Trop. Dis.* 3: e527.

**Saavedra-Rodriguez, K., C. L. Campbell, A. Lenhart, P. Penilla, S. Lozano-Fuentes, and W. C. Black IV. 2019.** Exome-wide association of deltamethrin resistance in *Aedes aegypti* from Mexico. *Insect Mol. Biol.* 28: 591-604.

**Saavedra-Rodriguez, K., L. Urdaneta-Marquez, S. Rajatileka, M. Moulton, A. E. Flores, I. Fernandez-Salas, J. Bisset, M. Rodriguez, P. J. McCall, M. J. Donnelly, H. Ranson, J. Hemingway, and W. C. Black IV. 2007.** A mutation in the voltage-gated sodium channel gene associated with pyrethroid resistance in Latin American *Aedes aegypti*. *Insect Mol. Biol.* 16: 785-798.
